# An updated checklist of the marine fish fauna of Redang Islands, Malaysia

**DOI:** 10.3897/BDJ.7.e47537

**Published:** 2019-12-06

**Authors:** Jianguo Du, Kar-Hoe Loh, Wenjia Hu, Xinqing Zheng, Yang Amri Affendi, Jillian Lean Sim Ooi, Zhiyuan Ma, Mohammed Rizman-Idid, Albert Apollo Chan

**Affiliations:** 1 Third Institute of Oceanography, Ministry of Natural Resources, Xiamen, 361005, China Third Institute of Oceanography, Ministry of Natural Resources Xiamen, 361005 China; 2 Fujian Provincial Station for Field Observation and Research of Island and Coastal Zone in Zhangzhou, Xiamen, 361005, China Fujian Provincial Station for Field Observation and Research of Island and Coastal Zone in Zhangzhou Xiamen, 361005 China; 3 Fujian Provincial Key Laboratory of Marine Ecological Conservation and Restoration, Xiamen, 361005, China Fujian Provincial Key Laboratory of Marine Ecological Conservation and Restoration Xiamen, 361005 China; 4 Institute of Ocean and Earth Sciences, University of Malaya, Kuala Lumpur, 50603, Malaysia Institute of Ocean and Earth Sciences, University of Malaya Kuala Lumpur, 50603 Malaysia; 5 Department of Geography, Faculty of Arts and Social Sciences, University of Malaya, Kuala Lumpur, 50603, Malaysia Department of Geography, Faculty of Arts and Social Sciences, University of Malaya Kuala Lumpur, 50603 Malaysia; 6 Marine Park and Resource Management Division, Department of Fisheries Malaysia, Putrajaya, 62628, Malaysia Marine Park and Resource Management Division, Department of Fisheries Malaysia Putrajaya, 62628 Malaysia

**Keywords:** Coral Fish Diversity Index, fish diversity, ichthyofauna, new record species

## Abstract

**Background:**

Redang Islands Marine Park consists of nine islands in the state of Terengganu, Malaysia. Redang Island is one of the largest off the east coast of Peninsular Malaysia, which is famous for its crystal-clear waters and white sandy beaches. The ichthyofauna of the Redang archipelago was surveyed by underwater visual observations between August 2016 and May 2018. Census data were compiled with existing records into the checklist of the marine fish of the Redang archipelago presented herein. A total of 314 species belonging to 51 families were recorded. The most speciose families (Pomacentridae, Labridae, Scaridae, Serranidae, Apogonidae, Carangidae, Gobiidae, Chaetodontidae, Lutjanidae, Nemipteridae and Siganidae) were also amongst the most speciose at the neighbouring Tioman archipelago (except Chaetodontidae). The coral fish diversity index value for the six families of coral reef fishes (Chaetodontidae, Pomacanthidae, Pomacentridae, Labridae, Scaridae and Acanthuridae) of the study sites was 132. We estimated that there were 427 coral reef fish species in the Redang archipelago. According to the IUCN Red List, eight species are Near Threatened (*Carcharhinus melanopterus*, *Chaetodon trifascialis*, *Choerodon schoenleinii*, *Epinephelus fuscoguttatus*, *E. polyphekadion*, *Plectropomus leopardus*, *Taeniura lymma* and *Triaenodon obesus*), eleven are Vulnerable (*Bolbometopon muricatum*, *Chaetodon trifasciatus, Chlorurus sordidus, Dascyllus trimaculatus, Epinephelus fuscoguttatus, E. polyphekadion, Halichoeres marginatus, Heniochus acuminatus, Nebrius ferrugineus, Neopomacentrus cyanomos* and *Plectropomus areolatus*) and three are Endangered (*Amphiprion clarkia, Cheilinus undulatus* and *Scarus ghobban*) in the Redang archipelago.

**New information:**

Five species are new records for Malaysia (*Ctenogobiops mitodes*, *Epibulus brevis*, *Halichoeres erdmanni*, *H. richmondi* and *Scarus caudofasciatus*) and 25 species are newly recorded in the Redang archipelago.

## Introduction

**Figure 1.**
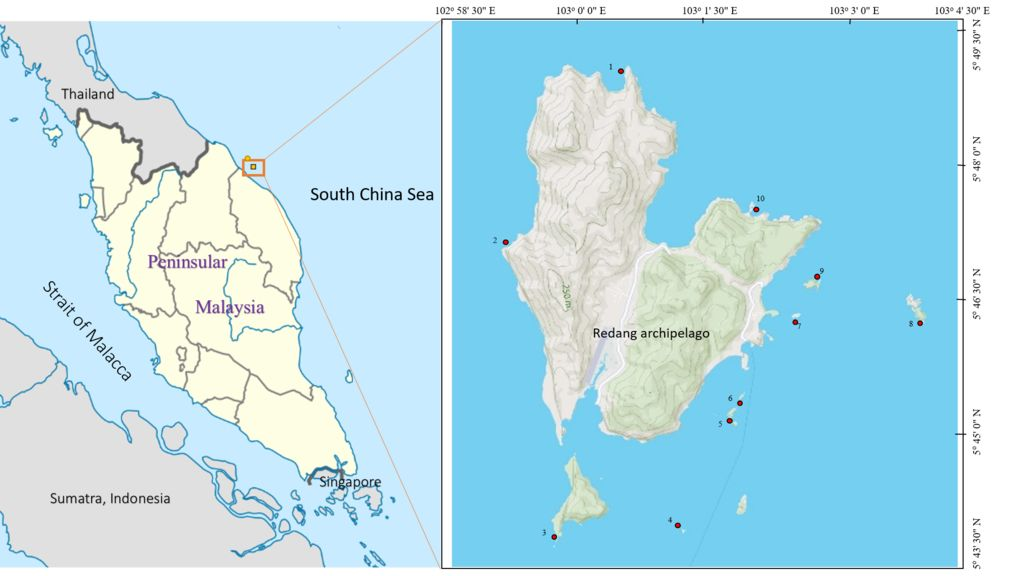
The map showing the location of Redang archipelago in the north Peninsular Malaysia, the inset map showing the survey locations.

Redang archipelago is located about 45 km to the northeast of Kuala Terengganu and it has a surface area of about 2,484 ha, making it the largest of the nine islands within the Redang archipelago. The Redang archipelago comprises the islands of Redang, Pinang, Ling, Ekor Tebu, Kerengga Besar, Kerengga Kechil, Paku Besar, Paku Kechil and Lima (Fig. 1). The waters surrounding the Redang archipelago have been protected as a Marine Park under the Establishment of Marine Park Malaysia Order 1994 (Fisheries Act in 1995) Comley et al. 2004. It is famous for its crystal-clear waters, white sandy beaches and for being an important conservation site for sea turtles.

The coral reefs of the Redang Islands are amongst the best on the east coast of Malaysia and are generally in good condition. Reef Check Malaysia has established an annual survey programme in Redang to assess the health of coral reefs since 2011. In 2016, the study showed that the reefs around Redang Islands are considered to be in “Good” condition, with live coral cover of 55.42%, which is above the average of 43.71% for reefs in Malaysia (Reef Check Malaysia 2016). Nevertheless, only the indicator fish species were monitored, for example, butterflyfish (targeted for the aquarium trade), humphead wrasse (live-food fish), snapper and grouper (food fish) (Reef Check Malaysia 2017).

A total of 209 species and 92 genera from 40 families of fish were reported from Redang Islands Marine Park by Harborne et al. (2000) and 173 species and 86 genera from 40 families of coral reef fish species were reported from the park by Yusuf et al. (2001). Furthermore, 441 marine and estuarine fish species and 108 families were recorded by Marsunuma et al. (2011) in the Kemaman, Dungun, Cendering, Kuala Terengganu, Merang and Kampung Raja fish landing ports or markets, mangroves at Setiu, Bidong Island and Redang Island. However, the fish list of the Redang Islands remains insufficient and there is an urgent need for taxonomic revisions, which can provide scientific data for the biodiversity of the marine fish fauna of the Redang Islands Marine Park.

## Materials and methods

A total of 10 sites in the Redang archipelago (Fig. 1) were investigated through underwater visual census in August 2016, May 2017, September 2017 and May 2018, with 30 dives altogether. The underwater cameras used were Canon 5D4, Canon G15 and Canon G1X Mark II, jointly with Sea&Sea YS-D2 flashlights. The coverage was limited to shallow reef fishes that are totally or mainly confined to coral reefs of < 30 m depth. The species were identified based on 600 high megapixel and high-quality photographs. Current taxonomic status follows that listed in the Catalog of Fishes by Fricke et al. (2018). The fish were identified based on: “Reef fishes of the east Indies” (Allen and Erdmann 2012), “Fishes of Terengganu, east coast of Malay Peninsula Malaysia” (Marsunuma et al. 2011) and FishBase (Froese and Pauly 2018).

The diversity of coral reef fish was estimated using the coral fish diversity index (CFDI) for restricted small areas as proposed by Allen (1988) and Allen and Erdmann (2012) using the following formula: total fish fauna = 3.39 (CFDI) – 20.595 for areas under 2,000 km^2^. This index was based on the total number of species in each of the six indicator families: Acanthuridae, Chaetodontidae, Labridae, Pomacanthidae, Pomacentridae and Scaridae. All selected groups are important components of reef communities, widely distributed and closely associated with coral reef ecosystems.

The checklist is arranged alphabetically by class, order and family and then species in families are arranged alphabetically by genera and then species name. The newly recorded species from Malaysia are marked with an asterisk (*) and species newly recorded in the Redang archipelago are marked with ±. The following abbreviations are used: standard length (SL); total length (TL); kilometres (km); hectares (ha); synonyms (s); misidentification (m). For habitats types: coastal shore (CS); shallow reef (SR); reef-associated (RFA), demersal (DEM); pelagic (PEL). Other remarks: Harborne et al. (2000):[2000]; Yusuf et al. (2001)[2001]; Marsunuma et al. (2011)[2011]; This study [2019]. The IUCN status (IUCN 2019): Endangered (EN); Near Threatened (NT); Vulnerable (VU). Threat to humans: Poisonous to eat; Reports of ciguatera poisoning; Traumatogenic; Venomous.

## Data resources

There were 140 species belonging to 36 families recorded in our survey, of which, five species represent new records in Malaysia (*Ctenogobiops mitodes*, *Epibulus brevis*, *Halichoeres erdmanni*, *H. richmondi* and *Scarus caudofasciatus*) and 25 species represent new records in the Redang archipelago (Table 1). Combined with data from previous sources, such as Harborne et al. (2000) and Yusuf et al. (2001), in total 314 RFA fish species were recorded from the Redang archipelago, belonging to 14 orders and 51 families. Most of the fishes (245 species) belonged to the order Perciformes. Pomacentridae (damselfish) was the most dominant family and had the most species (50 species), followed by Labridae (wrasses) with 44 species. Scaridae (parrotfish) and Serranidae (groupers) were the third largest families found in the area with 15 species. Other major families with high species richness were Apogonidae (cardinal fish), Carangidae (jacks or pompanos) and Gobiidae (gobies) with 14 species; Chaetodontidae (butterflyfish) with 13 species; Lutjanidae (snappers) and Siganidae (rabbitfishes) with 11 species; and Nemipteridae (threadfin breams) with 10 species (Table 1).

**Newly recorded species notes (Fig. 2)**

*Ctenogobiops mitodes* Randall, Shao & Chen, 2007 (Fig. 2a)

Common name: Thread shrimpgoby.

Maximum size of 5.3 cm SL (Froese and Pauly 2018). Characterised by whitish body colour with four longitudinal rows of dark brown spots, midlateral row largest and horizontally elongate; cheeks with row of three dark spots; behind lower edge of eye with oblique blue and yellow mark followed by similar marks on opercle and preopercle; curved blue and yellow line/dashes from behind upper eye to below dorsal fin origin; lower part of pectoral fin with elliptical white spot (Allen and Erdmann 2012). Western Pacific: Australia, Japan, Taiwan, South China Sea and Redang Island, Malaysia.

*Epibulus brevis* Carlson, Randall & Dawson, 2008 (Fig. 2b)

Common name: Latent slingjaw wrasse.

Maximum size of 18.5 cm SL (Froese and Pauly 2018). Brown or yellowish-brown with dark scale margins, yellow spot on back below base of third and fourth dorsal spines and yellow marking on posterior opercular flap. Females either dark brown or bright yellow with black scale margins and black pectoral fins; males dark brown to grey or dark greenish body, head at least partly green with no black stripe through eye; yellow band in lobes of caudal fin; no black on pectoral fins (Allen and Erdmann 2012). Western Pacific: Indonesia, Palau, Papua New Guinea, Philippines and Redang Island, Malaysia.

*Halichoeres erdmanni* Randall & Allen, 2010 (Fig. 2c)

Common name: Erdmann's wrasse.

Maximum size of 7.2 cm (male) and 5.9 cm (female) SL (Froese and Pauly 2018). Male greenish with reddish bands on head and combination of reddish stripes and irregular double bars on side; prominent black spot at pectoral base with yellow spot immediately behind; greenish spots and bands on reddish median fins and yellow-green zone at upper and lower rear edge of caudal fin. Female grey green, grading to white ventrally with about six red-orange stripes on side of body; black spot on anterior dorsal fin and smaller black spot on upper caudal-fin base (Allen and Erdmann 2012). Western Pacific: Berau Bay, Indonesia, west Papua, northern Gulf of Thailand, Singapore and Redang Island, Malaysia.

*Halichoeres richmondi* Fowler & Bean, 1928 (Fig. 2d)

Common name: Richmond's wrasse.

Maximum size of 19 cm (male) TL (Froese and Pauly 2018). Males yellowish-green with pale spots forming horizontal rows on body, head with broad red stripes and narrower blue stripes, yellow pectoral-fin base and purple-streaked blue caudal fin. Female yellow green with blue-green stripes on head and body, pair of black spots on dorsal fin and pale-edged black spot on upper caudal-fin base. Juveniles and females with orange to yellow anal fin. Snout more pointed compared with similar species (Allen and Erdmann 2012). Western Pacific: Java, Philippines, Ryukyu Islands, Molucas, Palau, Pohnpei, Kwajalein and Redang Island, Malaysia.

*Scaruscaudo fasciatus* (Günther, 1862) (Fig. 2e)

Common name: Red barred parrotfish.

Maximum size of 50 cm TL (Froese and Pauly 2018). Male green to blue green with pinkish scale margins, green band across end of snout continuing posteriorly under eye, green bands on chin and immediately below mouth; thin green stripe anterior to eye and continued for short distance behind eye; dorsal and anal fins reddish-pink with blue margin and dark-edged pinkish streak on each caudal lobe. Female blackish on anterior half with four alternating greyish and pale green to whitish bars on posterior half (Allen and Erdmann 2012). Western Indian Ocean, Maldives, Andaman Sea, and Redang Island, Malaysia

## Checklists

### Checklist of marine fishes of Redang archipelago

#### Acanthurus dussumieri

Valenciennes, 1835

566DB49A-F50A-5D82-880E-3F70B7BDD3A6

##### Materials

- **Type status:** Other material. **Occurrence:** occurrenceID: BDJ_12482_1; **Location:** country: Malaysia; locality: Redang islands; **Identification:** identifiedBy: Loh KH and Du Jianguo

##### Notes

Harborne et al. 2000

#### Acanthurus lineatus

(Linnaeus, 1758)

26C79CB1-86F4-59CD-B545-BFFFE3BB32AC

##### Materials

- **Type status:** Other material. **Occurrence:** occurrenceID: BDJ_12482_2; **Location:** country: Malaysia; locality: Redang islands; **Identification:** identifiedBy: Loh KH and Du Jianguo

##### Notes

Harborne et al. 2000

#### Acanthurus sp.

B600460C-88F1-573B-A219-3EFE5270C25E

##### Materials

- **Type status:** Other material. **Occurrence:** occurrenceID: BDJ_12482_3; **Location:** country: Malaysia; locality: Redang islands; **Identification:** identifiedBy: Yusuf YB, Mohd-Norizam M, Ali AB, Zaidnuddin I

##### Notes

Yusuf et al. 2001

#### Naso lituratus

(Forster, 1801)

63B810BF-144E-5A54-8CF8-C31D2D3F3039

##### Materials

- **Type status:** Other material. **Occurrence:** occurrenceID: BDJ_12482_4; **Location:** country: Malaysia; locality: Redang islands; **Identification:** identifiedBy: Loh KH and Du Jianguo

##### Notes

Harborne et al. 2000; Yusuf et al. 2001

#### Naso unicornis

(Forsskål, 1775)

78B64BAE-D390-58DB-A4FC-6DD5028F33AB

##### Materials

- **Type status:** Other material. **Occurrence:** occurrenceID: BDJ_12482_5; **Location:** country: Malaysia; locality: Redang islands; **Identification:** identifiedBy: Loh KH and Du Jianguo

##### Notes

Harborne et al. 2000

#### Apogon aureus

(Lacepède, 1802)

A00A9604-ACEE-52BD-B702-E270121C0B32

##### Materials

- **Type status:** Other material. **Occurrence:** occurrenceID: BDJ_12482_6; **Location:** country: Malaysia; locality: Redang islands; **Identification:** identifiedBy: Loh KH and Du Jianguo

##### Notes

Yusuf et al. 2001

#### Cheilodipterus artus

Smith, 1961

926FAB12-7464-52E7-9DA6-49A85A8D4FBE

##### Materials

- **Type status:** Other material. **Occurrence:** occurrenceID: BDJ_12482_7; **Location:** country: Malaysia; locality: Redang islands; **Identification:** identifiedBy: Loh KH and Du Jianguo

##### Notes

Harborne et al. 2000

#### Cheilodipterus intermedius

Gon, 1993

E679D0C5-81B3-57BB-977A-E7A9B55E7878

##### Materials

- **Type status:** Other material. **Occurrence:** occurrenceID: BDJ_12482_8; **Location:** country: Malaysia; locality: Redang islands; **Identification:** identifiedBy: Loh KH and Du Jianguo

##### Notes

Yusuf et al. 2001

#### Cheilodipterus macrodon

(Lacepède, 1802)

3CE75B62-7728-5439-A28D-0E46BCD1F3A2

##### Materials

- **Type status:** Other material. **Occurrence:** occurrenceID: BDJ_12482_9; **Location:** country: Malaysia; locality: Redang islands; **Identification:** identifiedBy: Loh KH and Du Jianguo

##### Notes

m: *Cheilodipterus heptaxona* Harborne et al. 2000; s: *Cheilodipterus microdon* Yusuf et al. 2001; This study.

#### Cheilodipterus quinquelineatus

Cuvier, 1828

8AFC5A6E-3221-500A-A152-20147C0785FD

##### Materials

- **Type status:** Other material. **Occurrence:** occurrenceID: BDJ_12482_10; **Location:** country: Malaysia; locality: Redang islands; **Identification:** identifiedBy: Loh KH and Du Jianguo

##### Notes

Harborne et al. 2000; Yusuf et al. 2001; This study.

#### Ostorhinchus chrysopomus

(Bleeker 1854)

7D9E9AB3-0E6E-5C08-A7B9-00016628BE59

##### Materials

- **Type status:** Other material. **Occurrence:** occurrenceID: BDJ_12482_11; **Location:** country: Malaysia; locality: Redang islands; **Identification:** identifiedBy: Loh KH and Du Jianguo

##### Notes

s: *Apogon crysopomus* Yusuf et al. 2001

#### Ostorhinchus compressus

(Smith & Radcliffe, 1911)

88DA86A1-9A39-59A2-B735-C165C7908D09

##### Materials

- **Type status:** Other material. **Occurrence:** occurrenceID: BDJ_12482_12; **Location:** country: Malaysia; locality: Redang islands; **Identification:** identifiedBy: Loh KH and Du Jianguo

##### Notes

Harborne et al. 2000; s: *Apogon compressus* Yusuf et al. 2001; This study.

#### Ostorhinchus cookii

(Macleay, 1881)

D6F143B7-0E01-54B1-9D52-7CBFE302E074

##### Materials

- **Type status:** Other material. **Occurrence:** occurrenceID: BDJ_12482_13; **Location:** country: Malaysia; locality: Redang islands; **Identification:** identifiedBy: Loh KH and Du Jianguo

##### Notes

s: *Ostorhinchus cookie* Harborne et al. 2000

#### Ostorhinchus cyanosoma

(Bleeker, 1853)

3746F663-349B-5356-94C0-65B2317F1B0E

##### Materials

- **Type status:** Other material. **Occurrence:** occurrenceID: BDJ_12482_14; **Location:** country: Malaysia; locality: Redang islands; **Identification:** identifiedBy: Loh KH and Du Jianguo

##### Notes

Harborne et al. 2000; s: *Apogon cyanosoma* Yusuf et al. 2001

#### Ostorhinchus wassinki

(Bleeker, 1860)

7D2D61B8-A8F8-593E-BF20-D169FB1BB756

##### Materials

- **Type status:** Other material. **Occurrence:** occurrenceID: BDJ_12482_15; **Location:** country: Malaysia; locality: Redang islands; **Identification:** identifiedBy: Loh KH and Du Jianguo

##### Notes

Newly recorded in Redang islands + This study

#### Pristicon rhodopterus

(Bleeker, 1852)

E6F4DAB8-CBF9-522B-AC7E-576C99749778

##### Materials

- **Type status:** Other material. **Occurrence:** occurrenceID: BDJ_12482_16; **Location:** country: Malaysia; locality: Redang islands; **Identification:** identifiedBy: Loh KH and Du Jianguo

##### Notes

s: *Apogon rhodopterus* Yusuf et al. 2001

#### Taeniamia fucata

(Cantor, 1849)

6DF92B99-5C3D-5947-9A2F-508BC2FD5704

##### Materials

- **Type status:** Other material. **Occurrence:** occurrenceID: BDJ_12482_17; **Location:** country: Malaysia; locality: Redang islands; **Identification:** identifiedBy: Loh KH and Du Jianguo

##### Notes

Harborne et al. 2000; s: *Archaima fucata* Yusuf et al. 2001; This study.

#### Taeniamia macroptera

(Cuvier, 1828)

D32DE2C5-30C9-5424-AB1E-5682C8FC1252

##### Materials

- **Type status:** Other material. **Occurrence:** occurrenceID: BDJ_12482_18; **Location:** country: Malaysia; locality: Redang islands; **Identification:** identifiedBy: Loh KH and Du Jianguo

##### Notes

s: *Archaima macroptera* Yusuf et al. 2001; This study.

#### Taeniamia zosterophora

(Bleeker, 1856)

700A9B7E-B60B-5F85-883E-FBDAA906CC93

##### Materials

- **Type status:** Other material. **Occurrence:** occurrenceID: BDJ_12482_19; **Location:** country: Malaysia; locality: Redang islands; **Identification:** identifiedBy: Loh KH and Du Jianguo

##### Notes

s: *Archamia zoesterophora* Harborne et al. (2000); s: *Cheilodipterus zosterophora* Yusuf et al. (2001); This study.

#### Balistapus undulatus

(Park, 1797)

179455DE-1212-53E9-97B5-3828C7D0F1E9

##### Materials

- **Type status:** Other material. **Occurrence:** occurrenceID: BDJ_12482_20; **Location:** country: Malaysia; locality: Redang islands; **Identification:** identifiedBy: Loh KH and Du Jianguo

##### Notes

Harborne et al. 2000

#### Balistoides viridescens

(Bloch & Schneider, 1801)

C96943C1-BA1C-55D2-84DF-9834F5666891

##### Materials

- **Type status:** Other material. **Occurrence:** occurrenceID: BDJ_12482_21; **Location:** country: Malaysia; locality: Redang islands; **Identification:** identifiedBy: Loh KH and Du Jianguo

##### Notes

Harborne et al. 2000; s: *Balistiodes viridescens* Yusuf et al. 2001

#### Melichthys vidua

(Richardson, 1845)

ABB934CB-6977-58FD-ADA3-86D93AB2D84F

##### Materials

- **Type status:** Other material. **Occurrence:** occurrenceID: BDJ_12482_22; **Location:** country: Malaysia; locality: Redang islands; **Identification:** identifiedBy: Loh KH and Du Jianguo

##### Notes

Yusuf et al. 2001

#### Pseudobalistes flavimarginatus

(Rüppell, 1829)

42E6B0F4-DA77-5B6F-B91E-1C4FF71562F2

##### Materials

- **Type status:** Other material. **Occurrence:** occurrenceID: BDJ_12482_23; **Location:** country: Malaysia; locality: Redang islands; **Identification:** identifiedBy: Loh KH and Du Jianguo

##### Notes

Harborne et al. 2000; Yusuf et al. 2001

#### Sufflamen bursa

(Bloch & Schneider, 1801)

BAA7855B-C3E6-5CEB-A321-63635B26DB96

##### Materials

- **Type status:** Other material. **Occurrence:** occurrenceID: BDJ_12482_24; **Location:** country: Malaysia; locality: Redang islands; **Identification:** identifiedBy: Loh KH and Du Jianguo

##### Notes

Harborne et al. 2000

#### Strongylura incisa

(Valenciennes, 1846)

9BBC2627-B46D-58CC-BF09-F9F4F1ECCDFF

##### Materials

- **Type status:** Other material. **Occurrence:** occurrenceID: BDJ_12482_25; **Location:** country: Malaysia; locality: Redang islands; **Identification:** identifiedBy: Loh KH and Du Jianguo

##### Notes

Harborne et al. 2000

#### Tylosurus crocodilus

(Péron & Lesueur, 1821)

E6064F83-33E1-50EC-B3DC-DA3317A6355C

##### Materials

- **Type status:** Other material. **Occurrence:** occurrenceID: BDJ_12482_26; **Location:** country: Malaysia; locality: Redang islands; **Identification:** identifiedBy: Loh KH and Du Jianguo

##### Notes

Yusuf et al. 2001

#### Aspidontus taeniatus

Quoy & Gaimard, 1834

DBE9CFF3-50B5-5509-B4DE-A3BD98718E9D

##### Materials

- **Type status:** Other material. **Occurrence:** occurrenceID: BDJ_12482_27; **Location:** country: Malaysia; locality: Redang islands; **Identification:** identifiedBy: Loh KH and Du Jianguo

##### Notes

Newly recorded in Redang islands + This study.

#### Atrosalarias fuscus

(Rüppell, 1838)

A3C30643-3B2A-5612-AFC2-DC52318778BC

##### Materials

- **Type status:** Other material. **Occurrence:** occurrenceID: BDJ_12482_28; **Location:** country: Malaysia; locality: Redang islands; **Identification:** identifiedBy: Loh KH and Du Jianguo

##### Notes

Harborne et al. 2000

#### Ecsenius bicolor

(Day, 1888)

97C69A38-DD49-5340-96B7-47205B38E0C0

##### Materials

- **Type status:** Other material. **Occurrence:** occurrenceID: BDJ_12482_29; **Location:** country: Malaysia; locality: Redang islands; **Identification:** identifiedBy: Loh KH and Du Jianguo

##### Notes

Harborne et al. 2000; This study.

#### Ecsenius lineatus

Klausewitz, 1962

69479ECD-972C-5EFD-9649-3FCF1F5CA883

##### Materials

- **Type status:** Other material. **Occurrence:** occurrenceID: BDJ_12482_30; **Location:** country: Malaysia; locality: Redang islands; **Identification:** identifiedBy: Loh KH and Du Jianguo

##### Notes

Harborne et al. 2000

#### Ecsenius yaeyamaensis

(Aoyagi, 1954)

D4D7A1CC-CBD9-551F-A1F6-AA0AFF26B1C7

##### Materials

- **Type status:** Other material. **Occurrence:** occurrenceID: BDJ_12482_31; **Location:** country: Malaysia; locality: Redang islands; **Identification:** identifiedBy: Loh KH and Du Jianguo

##### Notes

Harborne et al. 2000

#### Meiacanthus grammistes

(Valenciennes, 1836)

A70EF30C-E0E2-5B11-A406-ED2B1C5D3269

##### Materials

- **Type status:** Other material. **Occurrence:** occurrenceID: BDJ_12482_32; **Location:** country: Malaysia; locality: Redang islands; **Identification:** identifiedBy: Loh KH and Du Jianguo

##### Notes

Harborne et al. 2000

#### Plagoitremus rhinorhynchos

(Bleeker, 1852)

C9CC95A1-24AE-5579-BF81-BD08BB8B3C8E

##### Materials

- **Type status:** Other material. **Occurrence:** occurrenceID: BDJ_12482_33; **Location:** country: Malaysia; locality: Redang islands; **Identification:** identifiedBy: Loh KH and Du Jianguo

##### Notes

s: *Plagoitremus rhinorhincus* Yusuf et al. 2001

#### Salarias sp.

483073D2-68D1-5020-B159-4E5450AA75BC

##### Materials

- **Type status:** Other material. **Occurrence:** occurrenceID: BDJ_12482_34; **Location:** country: Malaysia; locality: Redang islands; **Identification:** identifiedBy: Yusuf YB, Mohd-Norizam M, Ali AB, Zaidnuddin I

##### Notes

Yusuf et al. 2001

#### Caesio caerulaurea

Lacepède, 1801

9DB63D04-4312-5D3C-B212-EFE99880488A

##### Materials

- **Type status:** Other material. **Occurrence:** occurrenceID: BDJ_12482_35; **Location:** country: Malaysia; locality: Redang islands; **Identification:** identifiedBy: Loh KH and Du Jianguo

##### Notes

Harborne et al. 2000; Yusuf et al. 2001; This study.

#### Caesio cuning

(Bloch, 1791)

2984CC0A-BA4F-5BFD-ADB9-A3CFB7F4A84A

##### Materials

- **Type status:** Other material. **Occurrence:** occurrenceID: BDJ_12482_36; **Location:** country: Malaysia; locality: Redang islands; **Identification:** identifiedBy: Loh KH and Du Jianguo

##### Notes

Harborne et al. 2000

#### Caesio teres

Seale, 1906

EA92F633-40F0-5315-8330-288BA59871F9

##### Materials

- **Type status:** Other material. **Occurrence:** occurrenceID: BDJ_12482_37; **Location:** country: Malaysia; locality: Redang islands; **Identification:** identifiedBy: Loh KH and Du Jianguo

##### Notes

Yusuf et al. 2001; This study.

#### Caesio xanthonota

Bleeker, 1853

DC77EF23-E5FB-57C1-8726-62FAD3CC8780

##### Materials

- **Type status:** Other material. **Occurrence:** occurrenceID: BDJ_12482_38; **Location:** country: Malaysia; locality: Redang islands; **Identification:** identifiedBy: Loh KH and Du Jianguo

##### Notes

s: *Caesio xanthonata* Yusuf et al. 2001

#### Pterocaesio chrysozona

(Cuvier, 1830)

036BD8E8-3F22-5DEC-9BFE-03D4A064127B

##### Materials

- **Type status:** Other material. **Occurrence:** occurrenceID: BDJ_12482_39; **Location:** country: Malaysia; locality: Redang islands; **Identification:** identifiedBy: Loh KH and Du Jianguo

##### Notes

Harborne et al. 2000

#### Pterocaesio marri

Schultz, 1953

E73CBA2F-D3FB-51AB-B4BE-8D66C4FA6CEA

##### Materials

- **Type status:** Other material. **Occurrence:** occurrenceID: BDJ_12482_40; **Location:** country: Malaysia; locality: Redang islands; **Identification:** identifiedBy: Loh KH and Du Jianguo

##### Notes

Harborne et al. 2000; This study.

#### Alepes melanoptera

(Swainson, 1839)

407B6BCD-FC9C-5ED4-9F27-28E9B629482C

##### Materials

- **Type status:** Other material. **Occurrence:** occurrenceID: BDJ_12482_41; **Location:** country: Malaysia; locality: Redang islands; **Identification:** identifiedBy: Loh KH and Du Jianguo

##### Notes

Newly recorded in Redang islands + This study

#### Alepes sp.

763FB4CC-A21D-5C0A-A23C-45CB0F33F43A

##### Materials

- **Type status:** Other material. **Occurrence:** occurrenceID: BDJ_12482_42; **Location:** country: Malaysia; locality: Redang islands; **Identification:** identifiedBy: Yusuf YB, Mohd-Norizam M, Ali AB, Zaidnuddin I

##### Notes

Yusuf et al. 2001

#### Carangoides bajad

(Forsskål, 1775)

C8A56818-D3BE-5FC1-A38C-85F67682C0B5

##### Materials

- **Type status:** Other material. **Occurrence:** occurrenceID: BDJ_12482_43; **Location:** country: Malaysia; locality: Redang islands; **Identification:** identifiedBy: Loh KH and Du Jianguo

##### Notes

Harborne et al. 2000; This study.

#### Carangoides chrysophrys

(Cuvier, 1833)

A4CCA337-FB6C-5A9A-B1F9-17364AF04FE2

##### Materials

- **Type status:** Other material. **Occurrence:** occurrenceID: BDJ_12482_44; **Location:** country: Malaysia; locality: Redang islands; **Identification:** identifiedBy: Loh KH and Du Jianguo

##### Notes

s: *Carangoides chrysophys* Harborne et al. 2000

#### Carangoides gymnostethus

(Cuvier, 1833)

FDB66AE5-35EC-56CA-89C6-5509ACB192F0

##### Materials

- **Type status:** Other material. **Occurrence:** occurrenceID: BDJ_12482_45; **Location:** country: Malaysia; locality: Redang islands; **Identification:** identifiedBy: Loh KH and Du Jianguo

##### Notes

Harborne et al. 2000

#### Caranx melampygus

Cuvier, 1833

23334AD0-CC8E-55C5-86DE-65417FF017AA

##### Materials

- **Type status:** Other material. **Occurrence:** occurrenceID: BDJ_12482_46; **Location:** country: Malaysia; locality: Redang islands; **Identification:** identifiedBy: Loh KH and Du Jianguo

##### Notes

Harborne et al. 2000

#### Caranx sexfasciatus

Quoy & Gaimard, 1825

D2F0C37A-CA1F-581B-BE84-844955801FC1

##### Materials

- **Type status:** Other material. **Occurrence:** occurrenceID: BDJ_12482_47; **Location:** country: Malaysia; locality: Redang islands; **Identification:** identifiedBy: Loh KH and Du Jianguo

##### Notes

Harborne et al. 2000

#### Caranx tille

Cuvier, 1833

786CDAF5-CC89-53DA-802C-537FC7D1C6A5

##### Materials

- **Type status:** Other material. **Occurrence:** occurrenceID: BDJ_12482_48; **Location:** country: Malaysia; locality: Redang islands; **Identification:** identifiedBy: Loh KH and Du Jianguo

##### Notes

Harborne et al. 2000

#### Elagatis bipinnulata

(Quoy & Gaimard, 1825)

BD603BE8-3DA1-5D85-900A-9D1991C34CC1

##### Materials

- **Type status:** Other material. **Occurrence:** occurrenceID: BDJ_12482_49; **Location:** country: Malaysia; locality: Redang islands; **Identification:** identifiedBy: Loh KH and Du Jianguo

##### Notes

Harborne et al. 2000

#### Gnathanodon speciosus

(Forsskål, 1775)

0FD2112A-C274-54D1-8CFB-7DE948D88972

##### Materials

- **Type status:** Other material. **Occurrence:** occurrenceID: BDJ_12482_50; **Location:** country: Malaysia; locality: Redang islands; **Identification:** identifiedBy: Loh KH and Du Jianguo

##### Notes

Harborne et al. 2000; Yusuf et al. 2001

#### Scomberoides lysan

(Forsskål, 1775)

2FBFF0FD-AC88-5598-A4CC-63C65961D984

##### Materials

- **Type status:** Other material. **Occurrence:** occurrenceID: BDJ_12482_51; **Location:** country: Malaysia; locality: Redang islands; **Identification:** identifiedBy: Loh KH and Du Jianguo

##### Notes

Yusuf et al. 2001

#### Selaroides leptolepis

(Cuvier, 1833)

9693891A-6463-5025-829D-CD6A0668984D

##### Materials

- **Type status:** Other material. **Occurrence:** occurrenceID: BDJ_12482_52; **Location:** country: Malaysia; locality: Redang islands; **Identification:** identifiedBy: Loh KH and Du Jianguo

##### Notes

Newly recorded in Redang islands + This study

#### Trachinotus bailloni

(Lacepède, 1801)

AAA5E0EB-7B49-5041-8773-BE8315BA1A28

##### Materials

- **Type status:** Other material. **Occurrence:** occurrenceID: BDJ_12482_53; **Location:** country: Malaysia; locality: Redang islands; **Identification:** identifiedBy: Loh KH and Du Jianguo

##### Notes

Harborne et al. 2000; Yusuf et al. 2001

#### Trachinotus blochii

(Lacepède, 1801)

1A64368F-D06C-5367-AD78-867CED26B4CC

##### Materials

- **Type status:** Other material. **Occurrence:** occurrenceID: BDJ_12482_54; **Location:** country: Malaysia; locality: Redang islands; **Identification:** identifiedBy: Loh KH and Du Jianguo

##### Notes

Harborne et al. 2000

#### Carcharhinus melanopterus

(Quoy & Gaimard, 1824)

A1B59E75-FD48-56AF-AE70-7D1C435A3257

##### Materials

- **Type status:** Other material. **Occurrence:** occurrenceID: BDJ_12482_55; **Location:** country: Malaysia; locality: Redang islands; **Identification:** identifiedBy: Loh KH and Du Jianguo

##### Ecological interactions

###### Conservation status

NT

##### Notes

Harborne et al. 2000; Yusuf et al. 2001;This study.

#### Triaenodon obesus

(Rüppell, 1837)

1359F177-2C75-55A2-B029-5A2557A7D5F5

##### Materials

- **Type status:** Other material. **Occurrence:** occurrenceID: BDJ_12482_56; **Location:** country: Malaysia; locality: Redang islands; **Identification:** identifiedBy: Loh KH and Du Jianguo

##### Ecological interactions

###### Conservation status

NT

##### Notes

Harborne et al. 2000

#### Aeoliscus strigatus

(Günther, 1861)

13AE8273-EBE0-5D94-81CC-3FFDA6AF03F7

##### Materials

- **Type status:** Other material. **Occurrence:** occurrenceID: BDJ_12482_57; **Location:** country: Malaysia; locality: Redang islands; **Identification:** identifiedBy: Loh KH and Du Jianguo

##### Notes

Yusuf et al. 2001

#### Chaetodon adiergastos

Seale, 1910

D95EA278-4B29-5B7B-A14E-664FE0E2FADA

##### Materials

- **Type status:** Other material. **Occurrence:** occurrenceID: BDJ_12482_58; **Location:** country: Malaysia; locality: Redang islands; **Identification:** identifiedBy: Loh KH and Du Jianguo

##### Notes

Harborne et al. 2000

#### Chaetodon auriga

Forsskål, 1775

1F73088D-B71B-5010-AC3C-D6BBF272B73C

##### Materials

- **Type status:** Other material. **Occurrence:** occurrenceID: BDJ_12482_59; **Location:** country: Malaysia; locality: Redang islands; **Identification:** identifiedBy: Loh KH and Du Jianguo

##### Notes

Harborne et al. 2000

#### Chaetodon baronessa

Cuvier, 1829

F74E7BF5-489B-5976-96E9-41B839EB466F

##### Materials

- **Type status:** Other material. **Occurrence:** occurrenceID: BDJ_12482_60; **Location:** country: Malaysia; locality: Redang islands; **Identification:** identifiedBy: Loh KH and Du Jianguo

##### Notes

Harborne et al. 2000; Yusuf et al. 2001; This study.

#### Chaetodon lineolatus

Cuvier, 1831

AFDB7A75-F47F-58D5-B3EF-E4DD4D6A01A5

##### Materials

- **Type status:** Other material. **Occurrence:** occurrenceID: BDJ_12482_61; **Location:** country: Malaysia; locality: Redang islands; **Identification:** identifiedBy: Loh KH and Du Jianguo

##### Notes

Harborne et al. 2000

#### Chaetodon lunula

(Lacepède, 1802)

203DFB62-AA40-5B81-A470-ADD7B939349A

##### Materials

- **Type status:** Other material. **Occurrence:** occurrenceID: BDJ_12482_62; **Location:** country: Malaysia; locality: Redang islands; **Identification:** identifiedBy: Loh KH and Du Jianguo

##### Notes

Yusuf et al. 2001

#### Chaetodon octofasciatus

Bloch, 1787

C733145C-F1F7-545C-867B-1EE1836862FC

##### Materials

- **Type status:** Other material. **Occurrence:** occurrenceID: BDJ_12482_63; **Location:** country: Malaysia; locality: Redang islands; **Identification:** identifiedBy: Loh KH and Du Jianguo

##### Notes

Harborne et al. 2000; Yusuf et al. 2001; This study.

#### Chaetodon trifascialis

Quoy & Gaimard, 1825

5B59B5D2-9941-5CBB-8314-EA7B5B0714F8

##### Materials

- **Type status:** Other material. **Occurrence:** occurrenceID: BDJ_12482_64; **Location:** country: Malaysia; locality: Redang islands; **Identification:** identifiedBy: Loh KH and Du Jianguo

##### Ecological interactions

###### Conservation status

NT

##### Notes

Harborne et al. 2000

#### Chaetodon trifasciatus

Park, 1797

AB1B6913-11B6-5560-BBFB-FDFFF27EF36F

##### Materials

- **Type status:** Other material. **Occurrence:** occurrenceID: BDJ_12482_65; **Location:** country: Malaysia; locality: Redang islands; **Identification:** identifiedBy: Loh KH and Du Jianguo

##### Notes

Harborne et al. 2000

#### Chaetodon wiebeli

Kaup, 1863

40FF044B-2945-5986-820A-AC226873241D

##### Materials

- **Type status:** Other material. **Occurrence:** occurrenceID: BDJ_12482_66; **Location:** country: Malaysia; locality: Redang islands; **Identification:** identifiedBy: Loh KH and Du Jianguo

##### Notes

Harborne et al. 2000; Yusuf et al. 2001

#### Chelmon rostratus

(Linnaeus, 1758)

4B065A02-A06C-5EFF-B25A-793F9F5C645F

##### Materials

- **Type status:** Other material. **Occurrence:** occurrenceID: BDJ_12482_67; **Location:** country: Malaysia; locality: Redang islands; **Identification:** identifiedBy: Loh KH and Du Jianguo

##### Notes

Harborne et al. 2000; This study; s: *Chelmon rostratum* Yusuf et al. 2001

#### Coradion chrysozonus

(Cuvier, 1831)

1469C6D7-2F25-5C2D-A713-68D7F5E9978D

##### Materials

- **Type status:** Other material. **Occurrence:** occurrenceID: BDJ_12482_68; **Location:** country: Malaysia; locality: Redang islands; **Identification:** identifiedBy: Loh KH and Du Jianguo

##### Notes

Harborne et al. 2000; Yusuf et al. 2001; This study.

#### Heniochus acuminatus

(Linnaeus, 1758)

C974413D-F760-5113-9164-44C5E3DDA2A5

##### Materials

- **Type status:** Other material. **Occurrence:** occurrenceID: BDJ_12482_69; **Location:** country: Malaysia; locality: Redang islands; **Identification:** identifiedBy: Loh KH and Du Jianguo

##### Notes

s: *Heniochus accuminatus* Harborne et al. 2000; s: *Heniochus acuminiatus* Yusuf et al. 2001

#### Heniochus varius

(Cuvier, 1829)

2F8BABC9-4C12-594A-B352-3EF19E3BCDE1

##### Materials

- **Type status:** Other material. **Occurrence:** occurrenceID: BDJ_12482_70; **Location:** country: Malaysia; locality: Redang islands; **Identification:** identifiedBy: Loh KH and Du Jianguo

##### Notes

Harborne et al. 2000

#### Chanos chanos

(Forsskål, 1775)

6E5826BC-929C-5F75-B095-6F2CDAA408DA

##### Materials

- **Type status:** Other material. **Occurrence:** occurrenceID: BDJ_12482_71; **Location:** country: Malaysia; locality: Redang islands; **Identification:** identifiedBy: Loh KH and Du Jianguo

##### Notes

Yusuf et al. 2001

#### Himantura sp.

985ED015-AAB3-5852-8A54-2B6364FB55B0

##### Materials

- **Type status:** Other material. **Occurrence:** occurrenceID: BDJ_12482_72; **Location:** country: Malaysia; locality: Redang islands; **Identification:** identifiedBy: Harborne A, Fenner D, Barnes A, Beger M, Harding S, Roxburgh T

##### Notes

Harborne et al. 2000

#### Taeniura lymma

(Forsskål, 1775)

58E34F11-967E-5C73-8818-8E33CE33BAFE

##### Materials

- **Type status:** Other material. **Occurrence:** occurrenceID: BDJ_12482_73; **Location:** country: Malaysia; locality: Redang islands; **Identification:** identifiedBy: Loh KH and Du Jianguo

##### Ecological interactions

###### Conservation status

NT

##### Notes

Harborne et al. 2000; Yusuf et al. 2001; This study.

#### Diodon hystrix

Linnaeus, 1758

FCB1D9E9-B733-5AD1-8F1D-D5B6216E700D

##### Materials

- **Type status:** Other material. **Occurrence:** occurrenceID: BDJ_12482_74; **Location:** country: Malaysia; locality: Redang islands; **Identification:** identifiedBy: Loh KH and Du Jianguo

##### Notes

Harborne et al. 2000; This study.

#### Diodon liturosus

Shaw, 1804

12DF47A4-5066-51A4-8B84-C0E81476CE03

##### Materials

- **Type status:** Other material. **Occurrence:** occurrenceID: BDJ_12482_75; **Location:** country: Malaysia; locality: Redang islands; **Identification:** identifiedBy: Loh KH and Du Jianguo

##### Notes

Harborne et al. 2000; This study.

#### Echeneis naucrates

Linnaeus, 1758

A3FB16F0-688E-5C94-BEB5-E6ED8AD8071C

##### Materials

- **Type status:** Other material. **Occurrence:** occurrenceID: BDJ_12482_76; **Location:** country: Malaysia; locality: Redang islands; **Identification:** identifiedBy: Loh KH and Du Jianguo

##### Notes

Harborne et al. 2000; This study.

#### Platax teira

(Forsskål, 1775)

2E14B142-A7F5-5B4A-9085-40A4F77A9154

##### Materials

- **Type status:** Other material. **Occurrence:** occurrenceID: BDJ_12482_77; **Location:** country: Malaysia; locality: Redang islands; **Identification:** identifiedBy: Loh KH and Du Jianguo

##### Notes

Harborne et al. 2000; Yusuf et al. 2001; This study.

#### Platax sp.

077A05A7-E7F0-5C00-9D6A-C1D19B30197D

##### Materials

- **Type status:** Other material. **Occurrence:** occurrenceID: BDJ_12482_78; **Location:** country: Malaysia; locality: Redang islands; **Identification:** identifiedBy: Harborne A, Fenner D, Barnes A, Beger M, Harding S, Roxburgh T

##### Notes

Harborne et al. 2000

#### Gerres oyena

(Forsskål, 1775)

4D61F44A-0C69-57B3-B4A1-3FF1B87F0A46

##### Materials

- **Type status:** Other material. **Occurrence:** occurrenceID: BDJ_12482_79; **Location:** country: Malaysia; locality: Redang islands; **Identification:** identifiedBy: Loh KH and Du Jianguo

##### Notes

Yusuf et al. 2001

#### Nebrius ferrugineus

(Lesson, 1831)

981D688D-6717-5C8E-884C-B8BCF8F61F91

##### Materials

- **Type status:** Other material. **Occurrence:** occurrenceID: BDJ_12482_80; **Location:** country: Malaysia; locality: Redang islands; **Identification:** identifiedBy: Loh KH and Du Jianguo

##### Ecological interactions

###### Conservation status

VU

##### Notes

Harborne et al. 2000

#### Diademichthys lineatus

(Sauvage, 1883)

27FF01EA-1710-5B84-95BA-960BD4BAFFA1

##### Materials

- **Type status:** Other material. **Occurrence:** occurrenceID: BDJ_12482_81; **Location:** country: Malaysia; locality: Redang islands; **Identification:** identifiedBy: Loh KH and Du Jianguo

##### Notes

Newly recorded in Redang islands + This study.

#### Amblyeleotris rubrimarginata

Mohlmann & Randall, 2002

D914D7F8-9E2E-5E51-9FC4-B3DCA4EEBDD4

##### Materials

- **Type status:** Other material. **Occurrence:** occurrenceID: BDJ_12482_82; **Location:** country: Malaysia; locality: Redang islands; **Identification:** identifiedBy: Loh KH and Du Jianguo

##### Notes

Newly recorded in Redang islands + This study.

#### Amblyeleotris steinitzi

(Klausewitz, 1974)

C837A454-2268-59E4-B596-4364259FF5DB

##### Materials

- **Type status:** Other material. **Occurrence:** occurrenceID: BDJ_12482_83; **Location:** country: Malaysia; locality: Redang islands; **Identification:** identifiedBy: Loh KH and Du Jianguo

##### Notes

Yusuf et al. 2001

#### Amblyeleotris phalaena

(Valenciennes, 1837)

474F4C85-1530-55CE-AF9B-4B417F1694C8

##### Materials

- **Type status:** Other material. **Occurrence:** occurrenceID: BDJ_12482_84; **Location:** country: Malaysia; locality: Redang islands; **Identification:** identifiedBy: Loh KH and Du Jianguo

##### Notes

Yusuf et al. 2001

#### Cryptocentrus cinctus

(Herre, 1936)

867655F2-1956-5FBE-BC99-8DF8DE640F8A

##### Materials

- **Type status:** Other material. **Occurrence:** occurrenceID: BDJ_12482_85; **Location:** country: Malaysia; locality: Redang islands; **Identification:** identifiedBy: Loh KH and Du Jianguo

##### Notes

Harborne et al. 2000; Yusuf et al. 2001

#### Ctenogobiops pomastictus

Lubbock & Polunin, 1977

4C93AB20-146B-5888-AF9D-B3B9ABAA6016

##### Materials

- **Type status:** Other material. **Occurrence:** occurrenceID: BDJ_12482_86; **Location:** country: Malaysia; locality: Redang islands; **Identification:** identifiedBy: Loh KH and Du Jianguo

##### Notes

s: *Ctenogobius pomastictus* Yusuf et al. (2001)

#### Ctenogobiops mitodes

Randall, Shao & Chen, 2007

F34F397C-BF3F-5B1A-ADE1-70C4D3E0081A

##### Materials

- **Type status:** Other material. **Occurrence:** occurrenceID: BDJ_12482_87; **Location:** country: Malaysia; locality: Redang islands; **Identification:** identifiedBy: Loh KH and Du Jianguo

##### Notes

* Newly recorded in Malaysia + This study.

#### Eviota sebreei

Jordan & Seale, 1906

AA08DA5F-5404-582B-8C3D-F5C1E2F9A2C5

##### Materials

- **Type status:** Other material. **Occurrence:** occurrenceID: BDJ_12482_88; **Location:** country: Malaysia; locality: Redang islands; **Identification:** identifiedBy: Loh KH and Du Jianguo

##### Notes

Newly recorded in Redang islands + This study.

#### Exyrias bellissimus

(Smith, 1959)

EF5B8D21-14BB-5F25-9988-727CA304AF22

##### Materials

- **Type status:** Other material. **Occurrence:** occurrenceID: BDJ_12482_89; **Location:** country: Malaysia; locality: Redang islands; **Identification:** identifiedBy: Loh KH and Du Jianguo

##### Notes

Newly recorded in Redang islands + This study.

#### Istigobius decoratus

(Herre, 1927)

99C7EFA9-159F-5E5B-8EA1-F714ACA2F6CF

##### Materials

- **Type status:** Other material. **Occurrence:** occurrenceID: BDJ_12482_90; **Location:** country: Malaysia; locality: Redang islands; **Identification:** identifiedBy: Loh KH and Du Jianguo

##### Notes

Yusuf et al. 2001

#### Istigobius goldmanni

(Bleeker, 1852)

9F14BFAD-D344-5197-B186-F2C0DF896969

##### Materials

- **Type status:** Other material. **Occurrence:** occurrenceID: BDJ_12482_91; **Location:** country: Malaysia; locality: Redang islands; **Identification:** identifiedBy: Loh KH and Du Jianguo

##### Notes

This study.

#### Istigobius nigroocellatus

(Günther, 1873)

95993804-0740-5D55-A68F-DC7B657AFFB3

##### Materials

- **Type status:** Other material. **Occurrence:** occurrenceID: BDJ_12482_92; **Location:** country: Malaysia; locality: Redang islands; **Identification:** identifiedBy: Loh KH and Du Jianguo

##### Notes

This study.

#### Valenciennea longipinnis

(Lay & Bennett, 1839)

C3036AD8-6660-568C-967F-7B09B8F1E9A6

##### Materials

- **Type status:** Other material. **Occurrence:** occurrenceID: BDJ_12482_93; **Location:** country: Malaysia; locality: Redang islands; **Identification:** identifiedBy: Loh KH and Du Jianguo

##### Notes

Yusuf et al. 2001

#### Valenciennea muralis

(Valenciennes, 1837)

C8AC9680-8CC9-5C32-A80B-FDD3AFE9DD0C

##### Materials

- **Type status:** Other material. **Occurrence:** occurrenceID: BDJ_12482_94; **Location:** country: Malaysia; locality: Redang islands; **Identification:** identifiedBy: Loh KH and Du Jianguo

##### Notes

Yusuf et al. 2001

#### Valenciennea sexguttata

(Valenciennes, 1837)

6CA64BCA-4FB6-5DF2-A89E-032167A81E6A

##### Materials

- **Type status:** Other material. **Occurrence:** occurrenceID: BDJ_12482_95; **Location:** country: Malaysia; locality: Redang islands; **Identification:** identifiedBy: Loh KH and Du Jianguo

##### Notes

Yusuf et al. 2001; This study.

#### Plectorhinchus albovittatus

(Rüppell, 1838)

5B119C43-206F-5347-961E-8E0E93E5A1A6

##### Materials

- **Type status:** Other material. **Occurrence:** occurrenceID: BDJ_12482_96; **Location:** country: Malaysia; locality: Redang islands; **Identification:** identifiedBy: Loh KH and Du Jianguo

##### Notes

Harborne et al. 2000

#### Plectorhinchus chaetodonoides

Lacepède, 1801

174B90D1-7A67-5C22-B40E-3C28F13B4D90

##### Materials

- **Type status:** Other material. **Occurrence:** occurrenceID: BDJ_12482_97; **Location:** country: Malaysia; locality: Redang islands; **Identification:** identifiedBy: Loh KH and Du Jianguo

##### Notes

Newly recorded in Redang islands + This study.

#### Plectorhinchus chrysotaenia

(Bleeker, 1855)

3691E358-1895-5C0D-A055-F4AE1BB2651B

##### Materials

- **Type status:** Other material. **Occurrence:** occurrenceID: BDJ_12482_98; **Location:** country: Malaysia; locality: Redang islands; **Identification:** identifiedBy: Loh KH and Du Jianguo

##### Notes

s: *Plectorhinchus celebicusYusuf et al. 2001*

#### Plectorhinchus gibbosus

(Lacepède, 1802)

6745CCEC-B4F5-547A-9F60-6F7AD96D1702

##### Materials

- **Type status:** Other material. **Occurrence:** occurrenceID: BDJ_12482_99; **Location:** country: Malaysia; locality: Redang islands; **Identification:** identifiedBy: Loh KH and Du Jianguo

##### Notes

Harborne et al. 2000; Yusuf et al. 2001

#### Plectorhinchus lessonii

(Cuvier, 1830)

00703691-4210-5CAC-A955-23DE94526BED

##### Materials

- **Type status:** Other material. **Occurrence:** occurrenceID: BDJ_12482_100; **Location:** country: Malaysia; locality: Redang islands; **Identification:** identifiedBy: Loh KH and Du Jianguo

##### Notes

s: *Plectorhinchus lessoniHarborne et al. 2000*

#### Plectorhinchus vittatus

(Linnaeus, 1758)

3D887C4F-7BC0-55ED-9ECF-BE683C2F59B1

##### Materials

- **Type status:** Other material. **Occurrence:** occurrenceID: BDJ_12482_101; **Location:** country: Malaysia; locality: Redang islands; **Identification:** identifiedBy: Loh KH and Du Jianguo

##### Notes

Harborne et al. 2000

#### Hemiramphus sp.

C9EDE0FA-3A65-5377-8A2B-24B74939F7D6

##### Materials

- **Type status:** Other material. **Occurrence:** occurrenceID: BDJ_12482_102; **Location:** country: Malaysia; locality: Redang islands; **Identification:** identifiedBy: Yusuf YB, Mohd-Norizam M, Ali AB, Zaidnuddin I

##### Notes

Yusuf et al. 2001

#### Hemiramphus affinis

(Güther, 1866)

60E39A10-FDAE-5AEE-B395-3D40DE292C0E

##### Materials

- **Type status:** Other material. **Occurrence:** occurrenceID: BDJ_12482_103; **Location:** country: Malaysia; locality: Redang islands; **Identification:** identifiedBy: Loh KH and Du Jianguo

##### Notes

s: *Hyporhampus affinis* Yusuf et al. 2001

#### Myripristis hexagona

(Lacepède, 1802)

43FF50B4-9042-5886-82E3-F5EA0BAF515A

##### Materials

- **Type status:** Other material. **Occurrence:** occurrenceID: BDJ_12482_104; **Location:** country: Malaysia; locality: Redang islands; **Identification:** identifiedBy: Loh KH and Du Jianguo

##### Notes

Newly recorded in Redang islands + This study.

#### Myripristis kuntee

Valenciennes, 1831

379DC4B2-3E37-5F1A-A30C-D0CD77443F2C

##### Materials

- **Type status:** Other material. **Occurrence:** occurrenceID: BDJ_12482_105; **Location:** country: Malaysia; locality: Redang islands; **Identification:** identifiedBy: Loh KH and Du Jianguo

##### Notes

Yusuf et al. 2001

#### Myripristis murdjan

(Forsskål, 1775)

3B11BE9C-2F7A-5920-AED0-21B53F85C7EC

##### Materials

- **Type status:** Other material. **Occurrence:** occurrenceID: BDJ_12482_106; **Location:** country: Malaysia; locality: Redang islands; **Identification:** identifiedBy: Loh KH and Du Jianguo

##### Notes

Harborne et al. 2000

#### Sargocentron cornutum

(Bleeker, 1854)

3F0C1173-28AE-5154-B402-DBD8281AF9E4

##### Materials

- **Type status:** Other material. **Occurrence:** occurrenceID: BDJ_12482_107; **Location:** country: Malaysia; locality: Redang islands; **Identification:** identifiedBy: Loh KH and Du Jianguo

##### Notes

Newly recorded in Redang islands + This study.

#### Sargocentron rubrum

(Forsskål, 1775)

46F6AC27-9450-5258-AECF-2CD8B51A630F

##### Materials

- **Type status:** Other material. **Occurrence:** occurrenceID: BDJ_12482_108; **Location:** country: Malaysia; locality: Redang islands; **Identification:** identifiedBy: Loh KH and Du Jianguo

##### Notes

This study.

#### Sargocentron praslin

(Lacepède, 1802)

EE334BDF-7806-560B-A112-C3B0938BDF56

##### Materials

- **Type status:** Other material. **Occurrence:** occurrenceID: BDJ_12482_109; **Location:** country: Malaysia; locality: Redang islands; **Identification:** identifiedBy: Loh KH and Du Jianguo

##### Notes

Harborne et al. 2000

#### Sargocentron sp.

47199059-6C3E-5EB3-A70D-79627C870A97

##### Materials

- **Type status:** Holotype. **Occurrence:** occurrenceID: BDJ_12482_110; **Location:** country: Malaysia; locality: Redang islands; **Identification:** identifiedBy: Yusuf YB, Mohd-Norizam M, Ali AB, Zaidnuddin I

##### Notes

Yusuf et al. 2001

#### Kyphosus cinerascens

(Forsskål, 1775)

BC1A80FE-BBB0-5438-9788-4E67AAA9DB28

##### Materials

- **Type status:** Other material. **Occurrence:** occurrenceID: BDJ_12482_111; **Location:** country: Malaysia; locality: Redang islands; **Identification:** identifiedBy: Loh KH and Du Jianguo

##### Notes

Yusuf et al. 2001

#### Kyphosus sp.

F3647073-2BCF-54DF-9D9D-5B6DC52BFB85

##### Materials

- **Type status:** Other material. **Occurrence:** occurrenceID: BDJ_12482_112; **Location:** country: Malaysia; locality: Redang islands; **Identification:** identifiedBy: Harborne A, Fenner D, Barnes A, Beger M, Harding S, Roxburgh T

##### Notes

Harborne et al. 2000

#### Bodianus diana

(Lacepède, 1801)

6E51CDDC-0831-569D-A54F-8E33FE4AF19A

##### Materials

- **Type status:** Other material. **Occurrence:** occurrenceID: BDJ_12482_113; **Location:** country: Malaysia; locality: Redang islands; **Identification:** identifiedBy: Loh KH and Du Jianguo

##### Notes

Harborne et al. 2000

#### Bodianus mesothorax

(Bloch & Schneider, 1801)

6EEA82B5-D988-5715-B0A6-8B62EB608107

##### Materials

- **Type status:** Other material. **Occurrence:** occurrenceID: BDJ_12482_114; **Location:** country: Malaysia; locality: Redang islands; **Identification:** identifiedBy: Loh KH and Du Jianguo

##### Notes

Harborne et al. 2000; Yusuf et al. 2001

#### Cheilinus chlorourus

(Bloch, 1791)

9728CE17-46B0-5E74-8FAE-D70D5CE7EF6E

##### Materials

- **Type status:** Other material. **Occurrence:** occurrenceID: BDJ_12482_115; **Location:** country: Malaysia; locality: Redang islands; **Identification:** identifiedBy: Loh KH and Du Jianguo

##### Notes

Harborne et al. 2000; This study; s: *Cheilinus clorourus* Yusuf et al. 2001.

#### Cheilinus fasciatus

(Bloch, 1791)

165288D4-1211-59CB-AEF3-BEBCE66D39DD

##### Materials

- **Type status:** Other material. **Occurrence:** occurrenceID: BDJ_12482_116; **Location:** country: Malaysia; locality: Redang islands; **Identification:** identifiedBy: Loh KH and Du Jianguo

##### Notes

Harborne et al. 2000; Yusuf et al. 2001; This study.

#### Cheilinus trilobatus

Rüppell, 1834

2B9612C0-CAEC-5E10-8D38-4AC1A9BA7995

##### Materials

- **Type status:** Other material. **Occurrence:** occurrenceID: BDJ_12482_117; **Location:** country: Malaysia; locality: Redang islands; **Identification:** identifiedBy: Loh KH and Du Jianguo

##### Notes

Harborne et al. 2000; Yusuf et al. 2001; This study.

#### Cheilinus undulatus

Rüppell, 1835

8B9B03EF-4F9B-5C64-AACD-4A957951FCA8

##### Materials

- **Type status:** Other material. **Occurrence:** occurrenceID: BDJ_12482_118; **Location:** country: Malaysia; locality: Redang islands; **Identification:** identifiedBy: Loh KH and Du Jianguo

##### Notes

Yusuf et al. 2001

#### Choerodon schoenleinii

(Valenciennes, 1839)

C8C9EDEE-3D20-5820-9617-9A7ECE5CF2FA

##### Materials

- **Type status:** Other material. **Occurrence:** occurrenceID: BDJ_12482_119; **Location:** country: Malaysia; locality: Redang islands; **Identification:** identifiedBy: Loh KH and Du Jianguo

##### Notes

Harborne et al. 2000

#### Diproctacanthus xanthurus

(Bleeker, 1856)

D8374E67-56BD-563C-ABDD-D0E07262890A

##### Materials

- **Type status:** Other material. **Occurrence:** occurrenceID: BDJ_12482_120; **Location:** country: Malaysia; locality: Redang islands; **Identification:** identifiedBy: Loh KH and Du Jianguo

##### Notes

Harborne et al. 2000; Yusuf et al. 2001; This study.

#### Epibulus brevis

Carlson, Randall & Dawson, 2008

42D67F34-B2BD-5F99-82C5-5D4C46C1DC97

##### Materials

- **Type status:** Other material. **Occurrence:** occurrenceID: BDJ_12482_121; **Location:** country: Malaysia; locality: Redang islands; **Identification:** identifiedBy: Loh KH and Du Jianguo

##### Notes

* Newly recorded in Malaysia + This study.

#### Epibulus insidiator

(Pallas, 1770)

D77BE994-9D16-541F-ADCE-F88BA68043D2

##### Materials

- **Type status:** Other material. **Occurrence:** occurrenceID: BDJ_12482_122; **Location:** country: Malaysia; locality: Redang islands; **Identification:** identifiedBy: Loh KH and Du Jianguo

##### Notes

Harborne et al. 2000; Yusuf et al. 2001

#### Gomphosus varius

Lacepède, 1801

BB35E4F1-DCE7-5716-A32F-0C7E56674052

##### Materials

- **Type status:** Other material. **Occurrence:** occurrenceID: BDJ_12482_123; **Location:** country: Malaysia; locality: Redang islands; **Identification:** identifiedBy: Loh KH and Du Jianguo

##### Notes

Harborne et al. 2000; Yusuf et al. 2001; This study.

#### Halichoeres bicolor

(Bloch & Schneider, 1801)

0C3BA962-0FA2-5659-9926-DF46711B9430

##### Materials

- **Type status:** Other material. **Occurrence:** occurrenceID: BDJ_12482_124; **Location:** country: Malaysia; locality: Redang islands; **Identification:** identifiedBy: Loh KH and Du Jianguo

##### Notes

Newly recorded in Redang islands + This study.

#### Halichoeres biocellatus

Schultz, 1960

A0190C91-1405-5AEE-B771-DB8DB70F828D

##### Materials

- **Type status:** Other material. **Occurrence:** occurrenceID: BDJ_12482_125; **Location:** country: Malaysia; locality: Redang islands; **Identification:** identifiedBy: Loh KH and Du Jianguo

##### Notes

Harborne et al. 2000

#### Halichoeres chloropterus

(Bloch, 1791)

444BFAB0-9183-5D92-BAC9-8B16B7DFA2C1

##### Materials

- **Type status:** Other material. **Occurrence:** occurrenceID: BDJ_12482_126; **Location:** country: Malaysia; locality: Redang islands; **Identification:** identifiedBy: Loh KH and Du Jianguo

##### Notes

Harborne et al. 2000; Yusuf et al. 2001

#### Halichoeres dussumieri

(Bloch & Schneider, 1801)

0C9F9F4C-92F8-5C15-86A4-2C0CDB157569

##### Materials

- **Type status:** Other material. **Occurrence:** occurrenceID: BDJ_12482_127; **Location:** country: Malaysia; locality: Redang islands; **Identification:** identifiedBy: Loh KH and Du Jianguo

##### Notes

Yusuf et al. 2001

#### Halichoeres erdmanni

Randall & Allen, 2010

4A0C30D5-51E9-5252-B4F8-DA2E4AE1A031

##### Materials

- **Type status:** Other material. **Occurrence:** occurrenceID: BDJ_12482_128; **Location:** country: Malaysia; locality: Redang islands; **Identification:** identifiedBy: Loh KH and Du Jianguo

##### Notes

* Newly recorded in Malaysia + This study.

#### Halichoeres hortulanus

(Lacepède, 1801)

222CB471-262E-549F-A119-A666D8E75E81

##### Materials

- **Type status:** Other material. **Occurrence:** occurrenceID: BDJ_12482_129; **Location:** country: Malaysia; locality: Redang islands; **Identification:** identifiedBy: Loh KH and Du Jianguo

##### Notes

Yusuf et al. 2001; This study.

#### Halichoeres leucurus

(Walbaum, 1792)

6C3A9A19-3C48-56E5-80D3-F31AFDDC81F8

##### Materials

- **Type status:** Other material. **Occurrence:** occurrenceID: BDJ_12482_130; **Location:** country: Malaysia; locality: Redang islands; **Identification:** identifiedBy: Loh KH and Du Jianguo

##### Notes

s: *Halichoeres purpurascens* Harborne et al. 2000; This study.

#### Halichoeres marginatus

Rüppell, 1835

E06A8FE4-B180-5869-8821-40CDFB94875C

##### Materials

- **Type status:** Other material. **Occurrence:** occurrenceID: BDJ_12482_131; **Location:** country: Malaysia; locality: Redang islands; **Identification:** identifiedBy: Loh KH and Du Jianguo

##### Notes

Harborne et al. 2000; Yusuf et al. 2001

#### Halichoeres melanochir

Fowler & Bean, 1928

EB186D99-33AE-50A0-A8C0-63937D51A1BB

##### Materials

- **Type status:** Other material. **Occurrence:** occurrenceID: BDJ_12482_132; **Location:** country: Malaysia; locality: Redang islands; **Identification:** identifiedBy: Loh KH and Du Jianguo

##### Notes

This study.

#### Halichoeres melanurus

(Bleeker, 1851)

836944D0-F1D8-50D7-B87D-862ADDFB4588

##### Materials

- **Type status:** Other material. **Occurrence:** occurrenceID: BDJ_12482_133; **Location:** country: Malaysia; locality: Redang islands; **Identification:** identifiedBy: Loh KH and Du Jianguo

##### Notes

Harborne et al. 2000

#### Halichoeres nebulosus

(Valenciennes, 1839)

1385C23D-05BF-50F2-B52E-B167A72351F0

##### Materials

- **Type status:** Other material. **Occurrence:** occurrenceID: BDJ_12482_134; **Location:** country: Malaysia; locality: Redang islands; **Identification:** identifiedBy: Loh KH and Du Jianguo

##### Notes

Marsunuma et al. 2011; This study.

#### Halichoeres prosopeion

(Bleeker, 1853)

9D260234-ACA7-5C12-9010-03B8E6A05ED2

##### Materials

- **Type status:** Other material. **Occurrence:** occurrenceID: BDJ_12482_135; **Location:** country: Malaysia; locality: Redang islands; **Identification:** identifiedBy: Loh KH and Du Jianguo

##### Notes

This study.

#### Halichoeres richmondi

Fowler & Bean, 1928

C123B11E-BD3F-5BDB-BCC3-F78B27B6BC53

##### Materials

- **Type status:** Other material. **Occurrence:** occurrenceID: BDJ_12482_136; **Location:** country: Malaysia; locality: Redang islands; **Identification:** identifiedBy: Loh KH and Du Jianguo

##### Notes

* Newly recorded in Malaysia + This study.

#### Halichoeres scapularis

(Bennett, 1832)

B2621FAC-0DBB-54D2-BE47-8F538790DDBD

##### Materials

- **Type status:** Other material. **Occurrence:** occurrenceID: BDJ_12482_137; **Location:** country: Malaysia; locality: Redang islands; **Identification:** identifiedBy: Loh KH and Du Jianguo

##### Notes

Harborne et al. 2000; Yusuf et al. 2001

#### Halichoeres vrolikii

(Bleeker, 1855)

0C64095F-156F-5859-8E8E-1E3A39BDDBB9

##### Materials

- **Type status:** Other material. **Occurrence:** occurrenceID: BDJ_12482_138; **Location:** country: Malaysia; locality: Redang islands; **Identification:** identifiedBy: Loh KH and Du Jianguo

##### Notes

Harborne et al. 2000

#### Hemigymnus melapterus

(Bloch, 1791)

DC28331F-56B4-52C2-8B6A-93BFA1A9E51A

##### Materials

- **Type status:** Other material. **Occurrence:** occurrenceID: BDJ_12482_139; **Location:** country: Malaysia; locality: Redang islands; **Identification:** identifiedBy: Loh KH and Du Jianguo

##### Notes

Harborne et al. 2000; Yusuf et al. 2001; This study.

#### Labrichthys unilineatus

(Guichenot, 1847)

930B2FA2-0B16-51D2-8E34-9ED9C38820E5

##### Materials

- **Type status:** Other material. **Occurrence:** occurrenceID: BDJ_12482_140; **Location:** country: Malaysia; locality: Redang islands; **Identification:** identifiedBy: Loh KH and Du Jianguo

##### Notes

Harborne et al. 2000; Yusuf et al. 2001

#### Labroides dimidiatus

(Valenciennes, 1839)

CD01D4B5-6385-5A99-A075-C14D78845264

##### Materials

- **Type status:** Other material. **Occurrence:** occurrenceID: BDJ_12482_141; **Location:** country: Malaysia; locality: Redang islands; **Identification:** identifiedBy: Loh KH and Du Jianguo

##### Notes

Harborne et al. 2000; Yusuf et al. 2001; This study.

#### Leptojulis cyanopleura

(Bleeker, 1853)

672AD7F8-8D2C-5DA7-8483-184260555E6C

##### Materials

- **Type status:** Other material. **Occurrence:** occurrenceID: BDJ_12482_142; **Location:** country: Malaysia; locality: Redang islands; **Identification:** identifiedBy: Loh KH and Du Jianguo

##### Notes

Harborne et al. 2000

#### Macropharyngodon meleagris

(Valenciennes, 1839)

21152958-1C27-5917-BE4C-1D5ADA3A81CC

##### Materials

- **Type status:** Other material. **Occurrence:** occurrenceID: BDJ_12482_143; **Location:** country: Malaysia; locality: Redang islands; **Identification:** identifiedBy: Loh KH and Du Jianguo

##### Notes

Harborne et al. 2000

#### Oxycheilinus celebicus

(Bleeker, 1853)

58DB1C86-0EF3-5AB7-936E-BBE482EDEB56

##### Materials

- **Type status:** Other material. **Occurrence:** occurrenceID: BDJ_12482_144; **Location:** country: Malaysia; locality: Redang islands; **Identification:** identifiedBy: Loh KH and Du Jianguo

##### Notes

Harborne et al. 2000

#### Oxycheilinus digramma

(Lacepède, 1801)

1A043B11-1EB5-5023-B45A-183EBE795359

##### Materials

- **Type status:** Other material. **Occurrence:** occurrenceID: BDJ_12482_145; **Location:** country: Malaysia; locality: Redang islands; **Identification:** identifiedBy: Loh KH and Du Jianguo

##### Notes

Harborne et al. 2000; This study; s: *Oxychelinius diagrammusYusuf et al. 2001*.

#### Oxycheilinus mentalis

(Rüppell, 1828)

98BC70F3-44E0-5427-A38A-01DFFCFF90BA

##### Materials

- **Type status:** Other material. **Occurrence:** occurrenceID: BDJ_12482_146; **Location:** country: Malaysia; locality: Redang islands; **Identification:** identifiedBy: Loh KH and Du Jianguo

##### Notes

s: *Oxychelinius mentalis* Yusuf et al. 2001.

#### Oxycheilinus orientalis

(Günther, 1862)

919D7DA0-0AFA-5D4F-94B2-6EBE88AE3ACD

##### Materials

- **Type status:** Other material. **Occurrence:** occurrenceID: BDJ_12482_147; **Location:** country: Malaysia; locality: Redang islands; **Identification:** identifiedBy: Loh KH and Du Jianguo

##### Notes

Harborne et al. 2000

#### Oxycheilinus sp.

CDD287E0-FC10-5869-822C-958ACA518524

##### Materials

- **Type status:** Holotype. **Occurrence:** occurrenceID: BDJ_12482_148; **Location:** country: Malaysia; locality: Redang islands; **Identification:** identifiedBy: Yusuf YB, Mohd-Norizam M, Ali AB, Zaidnuddin I

##### Notes

s: *Oxychelinius* sp. Yusuf et al. 2001.

#### Paracheilinus filamentosus

Allen, 1974

3A573F3D-3999-527E-8D77-4CA86713AACF

##### Materials

- **Type status:** Other material. **Occurrence:** occurrenceID: BDJ_12482_149; **Location:** country: Malaysia; locality: Redang islands; **Identification:** identifiedBy: Loh KH and Du Jianguo

##### Notes

s: *Paracheilinus filamentousHarborne et al. 2000*.

#### Pseudocheilinus evanidus

Jordan & Evermann, 1903

51F94D2C-9F7F-5AB0-91CC-36C023BB856A

##### Materials

- **Type status:** Other material. **Occurrence:** occurrenceID: BDJ_12482_150; **Location:** country: Malaysia; locality: Redang islands; **Identification:** identifiedBy: Loh KH and Du Jianguo

##### Notes

s: *Pseudocheilinus evanides* Harborne et al. 2000.

#### Pteragogus cryptus

Randall, 1981

0CC361A6-05E9-5EBF-9FF8-020ADEA48C94

##### Materials

- **Type status:** Other material. **Occurrence:** occurrenceID: BDJ_12482_151; **Location:** country: Malaysia; locality: Redang islands; **Identification:** identifiedBy: Loh KH and Du Jianguo

##### Notes

Harborne et al. 2000; Yusuf et al. 2001

#### Stethojulis bandanensis

(Bleeker, 1851)

7E104605-22BD-5B06-B7B0-A6B6F7AA2371

##### Materials

- **Type status:** Other material. **Occurrence:** occurrenceID: BDJ_12482_152; **Location:** country: Malaysia; locality: Redang islands; **Identification:** identifiedBy: Loh KH and Du Jianguo

##### Notes

Harborne et al. 2000

#### Stethojulis interrupta

(Bleeker, 1851)

9E8CBCA3-0DC6-50DE-A729-25D5F38D95B1

##### Materials

- **Type status:** Other material. **Occurrence:** occurrenceID: BDJ_12482_153; **Location:** country: Malaysia; locality: Redang islands; **Identification:** identifiedBy: Loh KH and Du Jianguo

##### Notes

Newly recorded in Redang islands + This study.

#### Stethojulis trilineata

(Bloch & Schneider, 1801)

B0F3E659-E0EA-51BA-B861-43F570A98772

##### Materials

- **Type status:** Other material. **Occurrence:** occurrenceID: BDJ_12482_154; **Location:** country: Malaysia; locality: Redang islands; **Identification:** identifiedBy: Loh KH and Du Jianguo

##### Notes

Harborne et al. 2000; Yusuf et al. 2001; This study.

#### Thalassoma hardwicke

(Bennett, 1830)

439F9557-53D4-5DF7-ABCF-87008A3A6005

##### Materials

- **Type status:** Other material. **Occurrence:** occurrenceID: BDJ_12482_155; **Location:** country: Malaysia; locality: Redang islands; **Identification:** identifiedBy: Loh KH and Du Jianguo

##### Notes

Harborne et al. 2000

#### Thalassoma lunare

(Linnaeus, 1758)

A7C8625E-C69D-5D80-8D97-D7536DF50029

##### Materials

- **Type status:** Other material. **Occurrence:** occurrenceID: BDJ_12482_156; **Location:** country: Malaysia; locality: Redang islands; **Identification:** identifiedBy: Loh KH and Du Jianguo

##### Notes

Yusuf et al. 2001; This study.

#### Lethrinus erythropterus

Valenciennes, 1830

AA1457B3-5BAA-54BB-A389-91990BCF6F7C

##### Materials

- **Type status:** Other material. **Occurrence:** occurrenceID: BDJ_12482_157; **Location:** country: Malaysia; locality: Redang islands; **Identification:** identifiedBy: Loh KH and Du Jianguo

##### Notes

Harborne et al. 2000; Yusuf et al. 2001; This study.

#### Lethrinus microdon

Valenciennes, 1830

8EBD95DC-9A7A-5C56-AD89-E92B4F5E9125

##### Materials

- **Type status:** Other material. **Occurrence:** occurrenceID: BDJ_12482_158; **Location:** country: Malaysia; locality: Redang islands; **Identification:** identifiedBy: Loh KH and Du Jianguo

##### Notes

Harborne et al. 2000

#### Lethrinus olivaceus

Valenciennes, 1830

3EDE9FC4-F2DC-5AA4-9771-05B8375B31A9

##### Materials

- **Type status:** Other material. **Occurrence:** occurrenceID: BDJ_12482_159; **Location:** country: Malaysia; locality: Redang islands; **Identification:** identifiedBy: Loh KH and Du Jianguo

##### Notes

Harborne et al. 2000; Yusuf et al. 2001

#### Lethrinus ornatus

Valenciennes, 1830

92A814DA-C210-59BF-9FF4-F6464263A8E5

##### Materials

- **Type status:** Other material. **Occurrence:** occurrenceID: BDJ_12482_160; **Location:** country: Malaysia; locality: Redang islands; **Identification:** identifiedBy: Loh KH and Du Jianguo

##### Notes

Harborne et al. 2000

#### Lethrinus sp.

99173BF9-DAF4-55B0-A8CC-F2F4E0A398D2

##### Materials

- **Type status:** Holotype. **Occurrence:** occurrenceID: BDJ_12482_161; **Location:** country: Malaysia; locality: Redang islands; **Identification:** identifiedBy: Yusuf YB, Mohd-Norizam M, Ali AB, Zaidnuddin I

##### Notes

Yusuf et al. 2001

#### Lutjanus argentimaculatus

(Forsskål, 1775)

5D883818-B13C-57AA-A955-1AB740F3CD49

##### Materials

- **Type status:** Other material. **Occurrence:** occurrenceID: BDJ_12482_162; **Location:** country: Malaysia; locality: Redang islands; **Identification:** identifiedBy: Loh KH and Du Jianguo

##### Notes

Harborne et al. 2000; Yusuf et al. 2001

#### Lutjanus bohar

(Forsskål, 1775)

DF1BEA39-1A69-557C-ACB2-18FAB3EA219B

##### Materials

- **Type status:** Other material. **Occurrence:** occurrenceID: BDJ_12482_163; **Location:** country: Malaysia; locality: Redang islands; **Identification:** identifiedBy: Loh KH and Du Jianguo

##### Notes

Harborne et al. 2000

#### Lutjanus decussatus

(Cuvier, 1828)

BC65B14B-3FA2-51EE-BAB0-26E8D86482AF

##### Materials

- **Type status:** Other material. **Occurrence:** occurrenceID: BDJ_12482_164; **Location:** country: Malaysia; locality: Redang islands; **Identification:** identifiedBy: Loh KH and Du Jianguo

##### Notes

Yusuf et al. 2001; This study.

#### Lutjanus fulviflamma

(Forsskål, 1775)

E9982674-7ADA-5607-A4B5-9C19957F0CEA

##### Materials

- **Type status:** Other material. **Occurrence:** occurrenceID: BDJ_12482_165; **Location:** country: Malaysia; locality: Redang islands; **Identification:** identifiedBy: Loh KH and Du Jianguo

##### Notes

Harborne et al. 2000

#### Lutjanus johnii

(Bloch, 1792)/

8F22C5BA-3923-5502-BA1E-FBBFE586D9F8

##### Materials

- **Type status:** Other material. **Occurrence:** occurrenceID: BDJ_12482_166; **Location:** country: Malaysia; locality: Redang islands; **Identification:** identifiedBy: Loh KH and Du Jianguo

##### Notes

Harborne et al. 2000

#### Lutjanus kasmira

(Forsskål, 1775)

9B98CE1C-751A-5AF4-8BD1-3530F1BD586C

##### Materials

- **Type status:** Other material. **Occurrence:** occurrenceID: BDJ_12482_167; **Location:** country: Malaysia; locality: Redang islands; **Identification:** identifiedBy: Loh KH and Du Jianguo

##### Notes

Harborne et al. 2000

#### Lutjanus lutjanus

Bloch, 1790

960DD12C-F4F7-531E-A4E3-38406C691ED5

##### Materials

- **Type status:** Other material. **Occurrence:** occurrenceID: BDJ_12482_168; **Location:** country: Malaysia; locality: Redang islands; **Identification:** identifiedBy: Loh KH and Du Jianguo

##### Notes

Harborne et al. 2000; Yusuf et al. 2001; This study.

#### Lutjanus quinquelineatus

(Bloch, 1790)

0ED0AF62-8603-5CA6-84C8-6ACF58B4FF87

##### Materials

- **Type status:** Other material. **Occurrence:** occurrenceID: BDJ_12482_169; **Location:** country: Malaysia; locality: Redang islands; **Identification:** identifiedBy: Loh KH and Du Jianguo

##### Notes

Yusuf et al. 2001; This study.

#### Lutjanus russellii

(Bleeker, 1849)

52C3743C-22F6-56CB-B66B-FF9F0D567AD9

##### Materials

- **Type status:** Other material. **Occurrence:** occurrenceID: BDJ_12482_170; **Location:** country: Malaysia; locality: Redang islands; **Identification:** identifiedBy: Loh KH and Du Jianguo

##### Notes

s: *Lutjanus russelli* Yusuf et al. 2001. This study.

#### Lutjanus vitta

(Quoy & Gaimard, 1824)

38257AF9-E47B-5B0C-848E-2F809CA91057

##### Materials

- **Type status:** Other material. **Occurrence:** occurrenceID: BDJ_12482_171; **Location:** country: Malaysia; locality: Redang islands; **Identification:** identifiedBy: Loh KH and Du Jianguo

##### Notes

Harborne et al. 2000; Yusuf et al. 2001; This study.

#### Macolor niger

(Forsskål, 1775)

0DFB92A1-9814-50B3-8D8D-B66B4DDC134C

##### Materials

- **Type status:** Other material. **Occurrence:** occurrenceID: BDJ_12482_172; **Location:** country: Malaysia; locality: Redang islands; **Identification:** identifiedBy: Loh KH and Du Jianguo

##### Notes

Harborne et al. 2000; Yusuf et al. 2001

#### Gunnelichthys monostigma

Smith, 1958

7263DE6E-7BE7-56EB-B12B-C6529081821C

##### Materials

- **Type status:** Other material. **Occurrence:** occurrenceID: BDJ_12482_173; **Location:** country: Malaysia; locality: Redang islands; **Identification:** identifiedBy: Loh KH and Du Jianguo

##### Notes

Harborne et al. 2000

#### Aluterus monoceros

(Linnaeus, 1758)

35BFA819-61A1-5C34-94BA-F62318906DE4

##### Materials

- **Type status:** Other material. **Occurrence:** occurrenceID: BDJ_12482_174; **Location:** country: Malaysia; locality: Redang islands; **Identification:** identifiedBy: Loh KH and Du Jianguo

##### Notes

s: *Aluterus monoceres* Harborne et al. 2000. This study.

#### Aluterus scriptus

(Osbeck, 1765)

E3BE8EFF-D514-5E1B-AB70-11BB41673706

##### Materials

- **Type status:** Other material. **Occurrence:** occurrenceID: BDJ_12482_175; **Location:** country: Malaysia; locality: Redang islands; **Identification:** identifiedBy: Loh KH and Du Jianguo

##### Notes

Harborne et al. 2000. s: *Aluteres scriptus* Yusuf et al. 2001

#### Cantherhines dumerilii

(Hollard, 1854)

04B280BF-B2D0-56CB-8EAE-29BCCBC87328

##### Materials

- **Type status:** Other material. **Occurrence:** occurrenceID: BDJ_12482_176; **Location:** country: Malaysia; locality: Redang islands; **Identification:** identifiedBy: Loh KH and Du Jianguo

##### Notes

Harborne et al. 2000

#### Monodactylus argenteus

(Linnaeus, 1758)

52E81BFB-54B0-535A-9B2B-CDB2379B5FD6

##### Materials

- **Type status:** Other material. **Occurrence:** occurrenceID: BDJ_12482_177; **Location:** country: Malaysia; locality: Redang islands; **Identification:** identifiedBy: Loh KH and Du Jianguo

##### Notes

Harborne et al. 2000; This study; s: *Monodactylus argentiusYusuf et al. 2001*

#### Mulloidichthys flavolineatus

(Lacepède, 1801)

99F55880-1EF5-51BD-8BD1-7D9DAD11BAAE

##### Materials

- **Type status:** Other material. **Occurrence:** occurrenceID: BDJ_12482_178; **Location:** country: Malaysia; locality: Redang islands; **Identification:** identifiedBy: Loh KH and Du Jianguo

##### Notes

Harborne et al. 2000

#### Parupeneus ciliatus

(Lacepède, 1801)

5BD7357E-5187-5E1F-93ED-AB7FE24234F3

##### Materials

- **Type status:** Other material. **Occurrence:** occurrenceID: BDJ_12482_179; **Location:** country: Malaysia; locality: Redang islands; **Identification:** identifiedBy: Loh KH and Du Jianguo

##### Notes

Harborne et al. 2000

#### Parupeneus indicus

(Shaw, 1803)

3A920483-9370-5050-BB97-AC707F9EDED0

##### Materials

- **Type status:** Other material. **Occurrence:** occurrenceID: BDJ_12482_180; **Location:** country: Malaysia; locality: Redang islands; **Identification:** identifiedBy: Loh KH and Du Jianguo

##### Notes

Harborne et al. 2000

#### Upeneus tragula

Richardson, 1846

C0D7E423-16EC-5A2C-9B12-EB07E0D0AA4C

##### Materials

- **Type status:** Other material. **Occurrence:** occurrenceID: BDJ_12482_181; **Location:** country: Malaysia; locality: Redang islands; **Identification:** identifiedBy: Loh KH and Du Jianguo

##### Notes

Harborne et al. 2000; This study.

#### Crenemugil crenilabis

(Forsskål, 1775)

45560212-8BE5-5222-92D9-8CE6BD9C7D18

##### Materials

- **Type status:** Other material. **Occurrence:** occurrenceID: BDJ_12482_182; **Location:** country: Malaysia; locality: Redang islands; **Identification:** identifiedBy: Loh KH and Du Jianguo

##### Notes

Yusuf et al. 2001

#### Gymnothorax fimbriatus

(Bennett, 1832)

8C6F4613-67F8-54F1-ADE2-AA9DE7ED2608

##### Materials

- **Type status:** Other material. **Occurrence:** occurrenceID: BDJ_12482_183; **Location:** country: Malaysia; locality: Redang islands; **Identification:** identifiedBy: Loh KH and Du Jianguo

##### Notes

Newly recorded in Redang islands + This study.

#### Gymnothorax javanicus

Bloch & Schneider, 1801

F549830F-D84A-5FF1-90A5-510526FA7ACD

##### Materials

- **Type status:** Other material. **Occurrence:** occurrenceID: BDJ_12482_184; **Location:** country: Malaysia; locality: Redang islands; **Identification:** identifiedBy: Loh KH and Du Jianguo

##### Notes

Harborne et al. 2000; This study.

#### Pentapodus caninus

(Cuvier, 1830)

208A2CB7-F319-5CAC-90F9-35846EEDB3B7

##### Materials

- **Type status:** Other material. **Occurrence:** occurrenceID: BDJ_12482_185; **Location:** country: Malaysia; locality: Redang islands; **Identification:** identifiedBy: Loh KH and Du Jianguo

##### Notes

Harborne et al. 2000; Yusuf et al. 2001

#### Pentapodus emeryii

(Richardson, 1843)

DDFE3323-778C-5F45-83D2-B004CD1DECB5

##### Materials

- **Type status:** Other material. **Occurrence:** occurrenceID: BDJ_12482_186; **Location:** country: Malaysia; locality: Redang islands; **Identification:** identifiedBy: Loh KH and Du Jianguo

##### Notes

Harborne et al. 2000

#### Scolopsis affinis

Peters, 1877

F9759D48-556C-5F70-BC94-524D9723B7DE

##### Materials

- **Type status:** Other material. **Occurrence:** occurrenceID: BDJ_12482_187; **Location:** country: Malaysia; locality: Redang islands; **Identification:** identifiedBy: Loh KH and Du Jianguo

##### Notes

Harborne et al. 2000; Yusuf et al. 2001; This study.

#### Scolopsis bilineata

(Bloch, 1793)

B7D400D2-724D-5D15-BA46-F60970E33AA1

##### Materials

- **Type status:** Other material. **Occurrence:** occurrenceID: BDJ_12482_188; **Location:** country: Malaysia; locality: Redang islands; **Identification:** identifiedBy: Loh KH and Du Jianguo

##### Notes

Harborne et al. 2000; This study; s: *Scolopsis bilineatusYusuf et al. 2001*.

#### Scolopsis ciliata

(Lacepède, 1802)

A889998C-9F6D-56D5-A5CA-A1FE540A25C9

##### Materials

- **Type status:** Other material. **Occurrence:** occurrenceID: BDJ_12482_189; **Location:** country: Malaysia; locality: Redang islands; **Identification:** identifiedBy: Loh KH and Du Jianguo

##### Notes

Harborne et al. 2000; This study; s: *Scolopsis ciliates* Yusuf et al. 2001.

#### Scolopsis lineata

Quoy & Gaimard, 1824

46DAF6A9-01AE-5D4B-A900-8EA82D17C63E

##### Materials

- **Type status:** Other material. **Occurrence:** occurrenceID: BDJ_12482_190; **Location:** country: Malaysia; locality: Redang islands; **Identification:** identifiedBy: Loh KH and Du Jianguo

##### Notes

Harborne et al. 2000; This study; s: *Scolopsis lineatus* Yusuf et al. 2001.

#### Scolopsis margaritifer

(Cuvier, 1830)

0CC22475-5FE8-5A19-9C95-6316611BD2C7

##### Materials

- **Type status:** Other material. **Occurrence:** occurrenceID: BDJ_12482_191; **Location:** country: Malaysia; locality: Redang islands; **Identification:** identifiedBy: Loh KH and Du Jianguo

##### Notes

Harborne et al. 2000; This study; s: *Scolopsis margaritiferaYusuf et al. 2001*.

#### Scolopsis monogramma

(Cuvier, 1830)

9681601E-1B50-5440-A3F0-23D5F79DB7D1

##### Materials

- **Type status:** Other material. **Occurrence:** occurrenceID: BDJ_12482_192; **Location:** country: Malaysia; locality: Redang islands; **Identification:** identifiedBy: Loh KH and Du Jianguo

##### Notes

Harborne et al. 2000; Yusuf et al. 2001; This study.

#### Scolopsis trilineata

Kner, 1868

A149C6BF-2F1C-567F-A63F-5F2AF18FE22F

##### Materials

- **Type status:** Other material. **Occurrence:** occurrenceID: BDJ_12482_193; **Location:** country: Malaysia; locality: Redang islands; **Identification:** identifiedBy: Loh KH and Du Jianguo

##### Notes

s: *Scolopsis trilineatus* Yusuf et al. 2001

#### Scolopsis vosmeri

(Bloch, 1792)

F879FE0A-E2D2-5636-841C-5E5E368932B3

##### Materials

- **Type status:** Other material. **Occurrence:** occurrenceID: BDJ_12482_194; **Location:** country: Malaysia; locality: Redang islands; **Identification:** identifiedBy: Loh KH and Du Jianguo

##### Notes

Harborne et al. 2000; Yusuf et al. 2001; This study.

#### Ostracion cubicus

Linnaeus, 1758

2FB3DFA7-EF43-52EB-A37D-7BE7825219F7

##### Materials

- **Type status:** Other material. **Occurrence:** occurrenceID: BDJ_12482_195; **Location:** country: Malaysia; locality: Redang islands; **Identification:** identifiedBy: Loh KH and Du Jianguo

##### Notes

Harborne et al. 2000; Yusuf et al. 2001; This study.

#### Pempheris oualensis

Cuvier, 1831

83594D7C-4DC7-548B-A0D2-6582E0FF8F07

##### Materials

- **Type status:** Other material. **Occurrence:** occurrenceID: BDJ_12482_196; **Location:** country: Malaysia; locality: Redang islands; **Identification:** identifiedBy: Loh KH and Du Jianguo

##### Notes

Harborne et al. 2000; This study.

#### Pempheris schwenkii

Bleeker, 1855

8FFD4990-1106-5E94-852C-225062A26D7A

##### Materials

- **Type status:** Other material. **Occurrence:** occurrenceID: BDJ_12482_197; **Location:** country: Malaysia; locality: Redang islands; **Identification:** identifiedBy: Loh KH and Du Jianguo

##### Notes

Harborne et al. 2000

#### Pempheris vanicolensis

Cuvier, 1831

D5205C13-686C-5BE2-AF39-C34DAAD21D48

##### Materials

- **Type status:** Other material. **Occurrence:** occurrenceID: BDJ_12482_198; **Location:** country: Malaysia; locality: Redang islands; **Identification:** identifiedBy: Loh KH and Du Jianguo

##### Notes

Yusuf et al. 2001

#### Parapercis snyderi

Jordan & Starks, 1905

0CC7EA3F-C2A5-5C38-A412-8C950E7C51C4

##### Materials

- **Type status:** Other material. **Occurrence:** occurrenceID: BDJ_12482_199; **Location:** country: Malaysia; locality: Redang islands; **Identification:** identifiedBy: Loh KH and Du Jianguo

##### Notes

Harborne et al. 2000; This study.

#### Parapercis xanthozona

(Bleeker, 1849)

07FC432E-BCBC-5CDC-8666-8CC6CEEAD1CF

##### Materials

- **Type status:** Other material. **Occurrence:** occurrenceID: BDJ_12482_200; **Location:** country: Malaysia; locality: Redang islands; **Identification:** identifiedBy: Loh KH and Du Jianguo

##### Notes

Harborne et al. 2000; This study.

#### Chaetodontoplus mesoleucus

(Bloch, 1787)

B89C9FC9-EFD4-55B3-8BEF-8F1AFA973778

##### Materials

- **Type status:** Other material. **Occurrence:** occurrenceID: BDJ_12482_201; **Location:** country: Malaysia; locality: Redang islands; **Identification:** identifiedBy: Loh KH and Du Jianguo

##### Notes

Harborne et al. 2000; This study; s: *Chaetodontoplus mesoleucas* Yusuf et al. 2001.

#### Pomacanthus annularis

(Bloch, 1787)

C5E95700-0758-5F8C-9517-4DDE421EB889

##### Materials

- **Type status:** Other material. **Occurrence:** occurrenceID: BDJ_12482_202; **Location:** country: Malaysia; locality: Redang islands; **Identification:** identifiedBy: Loh KH and Du Jianguo

##### Notes

Harborne et al. 2000; Yusuf et al. 2001; This study.

#### Pomacanthus imperator

(Bloch, 1787)

3933E123-B3DD-5220-BB25-4E9D8AA21FC3

##### Materials

- **Type status:** Other material. **Occurrence:** occurrenceID: BDJ_12482_203; **Location:** country: Malaysia; locality: Redang islands; **Identification:** identifiedBy: Loh KH and Du Jianguo

##### Notes

Newly recorded in Redang islands + This study.

#### Pomacanthus sexstriatus

(Cuvier, 1831)

2E740B87-BD29-5F30-A030-B76DC6490BB9

##### Materials

- **Type status:** Other material. **Occurrence:** occurrenceID: BDJ_12482_204; **Location:** country: Malaysia; locality: Redang islands; **Identification:** identifiedBy: Loh KH and Du Jianguo

##### Notes

Harborne et al. 2000; Yusuf et al. 2001; This study.

#### Pomacanthus xanthometopon

(Bleeker, 1853)

80AFF9FA-58BA-5A41-BDC6-1B2E7ADB17EE

##### Materials

- **Type status:** Other material. **Occurrence:** occurrenceID: BDJ_12482_205; **Location:** country: Malaysia; locality: Redang islands; **Identification:** identifiedBy: Loh KH and Du Jianguo

##### Notes

Harborne et al. 2000

#### Abudefduf bengalensis

(Bloch, 1787)

84B3E479-BCBA-590B-A699-68912CB1C828

##### Materials

- **Type status:** Other material. **Occurrence:** occurrenceID: BDJ_12482_206; **Location:** country: Malaysia; locality: Redang islands; **Identification:** identifiedBy: Loh KH and Du Jianguo

##### Notes

Yusuf et al. 2001; This study.

#### Abudefduf notatus

(Day, 1870)

13A7E7CA-C8DC-5E87-9BAB-63CA63A66967

##### Materials

- **Type status:** Other material. **Occurrence:** occurrenceID: BDJ_12482_207; **Location:** country: Malaysia; locality: Redang islands; **Identification:** identifiedBy: Loh KH and Du Jianguo

##### Notes

Harborne et al. 2000; Yusuf et al. 2001

#### Abudefduf septemfasciatus

(Cuvier, 1830)

DC0A73AF-4962-5AE9-8E2A-767298D90703

##### Materials

- **Type status:** Other material. **Occurrence:** occurrenceID: BDJ_12482_208; **Location:** country: Malaysia; locality: Redang islands; **Identification:** identifiedBy: Loh KH and Du Jianguo

##### Notes

Harborne et al. 2000

#### Abudefduf sexfasciatus

(Lacepède, 1801)

FB641014-6488-5F0C-8DF0-B78CAAAD3044

##### Materials

- **Type status:** Other material. **Occurrence:** occurrenceID: BDJ_12482_209; **Location:** country: Malaysia; locality: Redang islands; **Identification:** identifiedBy: Loh KH and Du Jianguo

##### Notes

Yusuf et al. 2001; This study.

#### Abudefduf sordidus

(Forsskål, 1775)

B7CA143E-EFDF-5048-8617-02B3AE5732E1

##### Materials

- **Type status:** Other material. **Occurrence:** occurrenceID: BDJ_12482_210; **Location:** country: Malaysia; locality: Redang islands; **Identification:** identifiedBy: Loh KH and Du Jianguo

##### Notes

Harborne et al. 2000; This study 2019.

#### Abudefduf vaigiensis

(Quoy & Gaimard, 1825)

37B9A8E6-4ABC-5581-B177-B0CE9918E882

##### Materials

- **Type status:** Other material. **Occurrence:** occurrenceID: BDJ_12482_211; **Location:** country: Malaysia; locality: Redang islands; **Identification:** identifiedBy: Loh KH and Du Jianguo

##### Notes

Harborne et al. 2000; Yusuf et al. 2001; This study.

#### Amblyglyphidodon aureus

(Cuvier, 1830)

543E4EC4-D4E9-5235-A05C-2263F8CA9602

##### Materials

- **Type status:** Other material. **Occurrence:** occurrenceID: BDJ_12482_212; **Location:** country: Malaysia; locality: Redang islands; **Identification:** identifiedBy: Loh KH and Du Jianguo

##### Notes

Harborne et al. 2000; This study.

#### Amblyglyphidodon curacao

(Bloch, 1787)

91FB6E29-4080-552F-A9DE-96F183C92056

##### Materials

- **Type status:** Other material. **Occurrence:** occurrenceID: BDJ_12482_213; **Location:** country: Malaysia; locality: Redang islands; **Identification:** identifiedBy: Loh KH and Du Jianguo

##### Notes

Harborne et al. 2000; Yusuf et al. 2001 ; This study.

#### Amblyglyphidodon leucogaster

(Bleeker, 1847)

924A9C67-0DF7-577A-A60B-1C60450424FB

##### Materials

- **Type status:** Other material. **Occurrence:** occurrenceID: BDJ_12482_214; **Location:** country: Malaysia; locality: Redang islands; **Identification:** identifiedBy: Loh KH and Du Jianguo

##### Notes

Harborne et al. 2000; Yusuf et al. 2001; This study.

#### Amblyglyphidodon sp.

266EAACF-7B5A-5A36-B6E1-B7641BF44424

##### Materials

- **Type status:** Other material. **Occurrence:** occurrenceID: BDJ_12482_215; **Location:** country: Malaysia; locality: Redang islands

##### Notes

Yusuf et al. 2001

#### Amphiprion clarkii

(Bennett, 1830)

F2A3684D-CD5C-53A6-B0E7-080674C6697C

##### Materials

- **Type status:** Other material. **Occurrence:** occurrenceID: BDJ_12482_216; **Location:** country: Malaysia; locality: Redang islands; **Identification:** identifiedBy: Loh KH and Du Jianguo

##### Notes

Harborne et al. 2000; Yusuf et al. 2001; This study.

#### Amphiprion frenatus

Brevoort, 1856

6C24A576-F296-5137-BF2C-6BD70369E74B

##### Materials

- **Type status:** Other material. **Occurrence:** occurrenceID: BDJ_12482_217; **Location:** country: Malaysia; locality: Redang islands; **Identification:** identifiedBy: Loh KH and Du Jianguo

##### Notes

Harborne et al. 2000; Yusuf et al. 2001; This study.

#### Amphiprion melanopus

Bleeker, 1852

6E455211-F17E-59EC-9F04-99C9B92C9964

##### Materials

- **Type status:** Other material. **Occurrence:** occurrenceID: BDJ_12482_218; **Location:** country: Malaysia; locality: Redang islands; **Identification:** identifiedBy: Loh KH and Du Jianguo

##### Notes

Yusuf et al. 2001

#### Amphiprion ocellaris

Cuvier, 1830

C96BA250-54A8-59AA-AF85-3D5E601E4267

##### Materials

- **Type status:** Other material. **Occurrence:** occurrenceID: BDJ_12482_219; **Location:** country: Malaysia; locality: Redang islands; **Identification:** identifiedBy: Loh KH and Du Jianguo

##### Notes

Harborne et al. 2000; Yusuf et al. 2001; This study.

#### Amphiprion perideraion

Bleeker, 1855

9CD798F8-E9E5-5459-A8F8-16E4ED92A82A

##### Materials

- **Type status:** Other material. **Occurrence:** occurrenceID: BDJ_12482_220; **Location:** country: Malaysia; locality: Redang islands; **Identification:** identifiedBy: Loh KH and Du Jianguo

##### Notes

Harborne et al. 2000; This study; s: *Amphiprion periderion* Yusuf et al. 2001.

#### Cheiloprion labiatus

(Day, 1870)

188EEEEB-47DE-5BD6-9C00-27CCB9D96DBD

##### Materials

- **Type status:** Other material. **Occurrence:** occurrenceID: BDJ_12482_221; **Location:** country: Malaysia; locality: Redang islands; **Identification:** identifiedBy: Loh KH and Du Jianguo

##### Notes

Harborne et al. 2000; Yusuf et al. 2001

#### Chromis atripectoralis

Welander & Schultz, 1951

D092607D-9357-5E89-8585-60C1E9C1A4D8

##### Materials

- **Type status:** Other material. **Occurrence:** occurrenceID: BDJ_12482_222; **Location:** country: Malaysia; locality: Redang islands; **Identification:** identifiedBy: Loh KH and Du Jianguo

##### Notes

Harborne et al. 2000; This study; s: *Cromis atripectoralisYusuf et al. 2001*.

#### Chromis lepidolepis

Bleeker, 1877

A1AE582C-F202-5265-A681-FF35FAC1F430

##### Materials

- **Type status:** Other material. **Occurrence:** occurrenceID: BDJ_12482_223; **Location:** country: Malaysia; locality: Redang islands; **Identification:** identifiedBy: Loh KH and Du Jianguo

##### Notes

s: *Chromis lepidolepsisHarborne et al. 2000*

#### Chromis ternatensis

(Bleeker, 1856)

0FA9A792-2A0E-5F91-94B6-BC27447EC91F

##### Materials

- **Type status:** Other material. **Occurrence:** occurrenceID: BDJ_12482_224; **Location:** country: Malaysia; locality: Redang islands; **Identification:** identifiedBy: Loh KH and Du Jianguo

##### Notes

Harborne et al. 2000

#### Chromis viridis

(Cuvier, 1830)

ED98EEC1-90A8-586C-87D4-3AF42F8FA7A7

##### Materials

- **Type status:** Other material. **Occurrence:** occurrenceID: BDJ_12482_225; **Location:** country: Malaysia; locality: Redang islands; **Identification:** identifiedBy: Loh KH and Du Jianguo

##### Notes

Newly recorded in Redang islands + This study.

#### Chromis weberi

Fowler & Bean, 1928

64575482-1270-5E13-9193-D03EF98DCFC7

##### Materials

- **Type status:** Other material. **Occurrence:** occurrenceID: BDJ_12482_226; **Location:** country: Malaysia; locality: Redang islands; **Identification:** identifiedBy: Loh KH and Du Jianguo

##### Notes

s: *Chromis weberii* Harborne et al. 2000

#### Chrysiptera leucopoma

(Cuvier, 1830)

A17A5316-7682-528A-8514-EBCE9CFAD845

##### Materials

- **Type status:** Other material. **Occurrence:** occurrenceID: BDJ_12482_227; **Location:** country: Malaysia; locality: Redang islands; **Identification:** identifiedBy: Loh KH and Du Jianguo

##### Notes

Yusuf et al. 2001

#### Dascyllus reticulatus

(Richardson, 1846)

7A87492F-4AFB-5045-B19F-520F02EF92D5

##### Materials

- **Type status:** Other material. **Occurrence:** occurrenceID: BDJ_12482_228; **Location:** country: Malaysia; locality: Redang islands; **Identification:** identifiedBy: Loh KH and Du Jianguo

##### Notes

Harborne et al. 2000; This study; s: *Dascyllus reticulates* Yusuf et al. 2001.

#### Dascyllus trimaculatus

(Rüppell, 1829)

9F79F817-6CF1-5DAA-9E79-A579B56E9F7D

##### Materials

- **Type status:** Other material. **Occurrence:** occurrenceID: BDJ_12482_229; **Location:** country: Malaysia; locality: Redang islands; **Identification:** identifiedBy: Loh KH and Du Jianguo

##### Notes

Harborne et al. 2000; Yusuf et al. 2001; This study.

#### Dischistodus chrysopoecilus

(Bleeker, 1858)

4D00B292-82AB-5B21-BE75-121E71D86086

##### Materials

- **Type status:** Other material. **Occurrence:** occurrenceID: BDJ_12482_230; **Location:** country: Malaysia; locality: Redang islands; **Identification:** identifiedBy: Loh KH and Du Jianguo

##### Notes

s: *Dischistodus chrysopaecilusYusuf et al. 2001*.

#### Dischistodus melanotus

(Bleeker, 1858)

02F97D50-FFA0-5EB0-B884-714F6467026A

##### Materials

- **Type status:** Other material. **Occurrence:** occurrenceID: BDJ_12482_231; **Location:** country: Malaysia; locality: Redang islands; **Identification:** identifiedBy: Loh KH and Du Jianguo

##### Notes

Harborne et al. 2000; Yusuf et al. 2001; This study.

#### Dischistodus perspicillatus

(Cuvier, 1830)

964FB233-7554-5385-9E63-8B28C2DB8EAB

##### Materials

- **Type status:** Other material. **Occurrence:** occurrenceID: BDJ_12482_232; **Location:** country: Malaysia; locality: Redang islands; **Identification:** identifiedBy: Loh KH and Du Jianguo

##### Notes

Harborne et al. 2000; This study; s: *Dischistodus perspiciliatusYusuf et al. 2001*.

#### Hemiglyphidodon plagiometopon

(Bleeker, 1852)

20AEAEF2-5808-5FB2-9570-27DF9D478AEC

##### Materials

- **Type status:** Other material. **Occurrence:** occurrenceID: BDJ_12482_233; **Location:** country: Malaysia; locality: Redang islands; **Identification:** identifiedBy: Loh KH and Du Jianguo

##### Notes

s: *Hemiglyphidodon plagiometapon* Harborne et al. 2000; s: *Hemiglyphidodon plagiometopodon* Yusuf et al. 2001

#### Neoglyphidodon melas

(Valenciennes, 1830)

F1B46A66-05CE-5867-87C7-E89A97D4C183

##### Materials

- **Type status:** Other material. **Occurrence:** occurrenceID: BDJ_12482_234; **Location:** country: Malaysia; locality: Redang islands; **Identification:** identifiedBy: Loh KH and Du Jianguo

##### Notes

Harborne et al. 2000; Yusuf et al. 2001; This study.

#### Neoglyphidodon nigroris

(Cuvier, 1830)

9C82F375-AAE3-5B3E-8A4F-0DB78D13BBD5

##### Materials

- **Type status:** Other material. **Occurrence:** occurrenceID: BDJ_12482_235; **Location:** country: Malaysia; locality: Redang islands; **Identification:** identifiedBy: Loh KH and Du Jianguo

##### Notes

Yusuf et al. 2001

#### Neoglyphidodon oxyodon

(Bleeker, 1858)

FDFA1270-A5CD-5B31-AC7D-60654DC6DD44

##### Materials

- **Type status:** Other material. **Occurrence:** occurrenceID: BDJ_12482_236; **Location:** country: Malaysia; locality: Redang islands; **Identification:** identifiedBy: Loh KH and Du Jianguo

##### Notes

Harborne et al. 2000

#### Neoglyphidodon thoracotaeniatus

(Fowler & Bean, 1928)

F3776595-6203-58A1-989C-C83018779146

##### Materials

- **Type status:** Other material. **Occurrence:** occurrenceID: BDJ_12482_237; **Location:** country: Malaysia; locality: Redang islands; **Identification:** identifiedBy: Loh KH and Du Jianguo

##### Notes

Harborne et al. 2000

#### Neopomacentrus anabatoides

(Bleeker, 1847)

855A00B9-26E6-5578-85F1-8F5C909B493A

##### Materials

- **Type status:** Other material. **Occurrence:** occurrenceID: BDJ_12482_238; **Location:** country: Malaysia; locality: Redang islands; **Identification:** identifiedBy: Loh KH and Du Jianguo

##### Notes

Harborne et al. 2000; Yusuf et al. 2001

#### Neopomacentrus azysron

(Bleeker, 1877)

F84313C9-3C7A-5B7E-BE5A-F7DB13E383E1

##### Materials

- **Type status:** Other material. **Occurrence:** occurrenceID: BDJ_12482_239; **Location:** country: Malaysia; locality: Redang islands; **Identification:** identifiedBy: Loh KH and Du Jianguo

##### Notes

s: *Neopomacentrus azyros* Harborne et al. 2000

#### Neopomacentrus cyanomos

(Bleeker, 1856)

0399F0EF-1E72-5FD3-B65B-44AA6B42CA8E

##### Materials

- **Type status:** Other material. **Occurrence:** occurrenceID: BDJ_12482_240; **Location:** country: Malaysia; locality: Redang islands; **Identification:** identifiedBy: Loh KH and Du Jianguo

##### Notes

Harborne et al. 2000; This study; s: *Dischistodus perspiciliatus* Yusuf et al. 2001.

#### Neopomacentrus violascens

(Bleeker, 1848)

CFB5B8C4-391D-5835-9A99-5120F9219D12

##### Materials

- **Type status:** Other material. **Occurrence:** occurrenceID: BDJ_12482_241; **Location:** country: Malaysia; locality: Redang islands; **Identification:** identifiedBy: Loh KH and Du Jianguo

##### Notes

Yusuf et al. 2001.

#### Plectroglyphidodon lacrymatus

(Quoy & Gaimard, 1825)

073BF69E-C816-5B58-8863-EE65C755CE84

##### Materials

- **Type status:** Other material. **Occurrence:** occurrenceID: BDJ_12482_242; **Location:** country: Malaysia; locality: Redang islands; **Identification:** identifiedBy: Loh KH and Du Jianguo

##### Notes

Harborne et al. 2000; Yusuf et al. 2001

#### Pomacentrus alexanderae

Evermann & Seale, 1907

4FC3A4F7-D279-5857-B642-B20EDF3D5E88

##### Materials

- **Type status:** Other material. **Occurrence:** occurrenceID: BDJ_12482_243; **Location:** country: Malaysia; locality: Redang islands; **Identification:** identifiedBy: Loh KH and Du Jianguo

##### Notes

Harborne et al. 2000; This study;. s: *Pomacentrus alexandraeYusuf et al. 2001*.

#### Pomacentrus armillatus

Allen, 1993

9DEECE8C-F17D-543C-B5C8-EB375162E33C

##### Materials

- **Type status:** Other material. **Occurrence:** occurrenceID: BDJ_12482_244; **Location:** country: Malaysia; locality: Redang islands; **Identification:** identifiedBy: Loh KH and Du Jianguo

##### Notes

Newly recorded in Redang islands + This study.

#### Pomacentrus bankanensis

Bleeker, 1854

9B216C8F-BD96-57EC-9A8C-842C8C5B1CBA

##### Materials

- **Type status:** Other material. **Occurrence:** occurrenceID: BDJ_12482_245; **Location:** country: Malaysia; locality: Redang islands; **Identification:** identifiedBy: Loh KH and Du Jianguo

##### Notes

Yusuf et al. 2001.

#### Pomacentrus bintanensis

Allen, 1999

25C1601E-157E-5118-B6F6-9B2B00A12F52

##### Materials

- **Type status:** Other material. **Occurrence:** occurrenceID: BDJ_12482_246; **Location:** country: Malaysia; locality: Redang islands; **Identification:** identifiedBy: Loh KH and Du Jianguo

##### Notes

Yusuf et al. 2001.

#### Pomacentrus chrysurus

Cuvier, 1830

B775068E-8603-58D6-A743-759F9FDED356

##### Materials

- **Type status:** Other material. **Occurrence:** occurrenceID: BDJ_12482_247; **Location:** country: Malaysia; locality: Redang islands; **Identification:** identifiedBy: Loh KH and Du Jianguo

##### Notes

Harborne et al. 2000; Yusuf et al. 2001.

#### Pomacentrus coelestis

Jordan & Starks, 1901

CEDB0F31-83D3-53A7-920C-AC7FF4707555

##### Materials

- **Type status:** Other material. **Occurrence:** occurrenceID: BDJ_12482_248; **Location:** country: Malaysia; locality: Redang islands; **Identification:** identifiedBy: Loh KH and Du Jianguo

##### Notes

Harborne et al. 2000; This study; s: *Pomacentrus coelestris* Yusuf et al. 2001.

#### Pomacentrus grammorhynchus

Fowler, 1918

CA51720E-C341-5A5F-8E65-6115CB90AA8E

##### Materials

- **Type status:** Other material. **Occurrence:** occurrenceID: BDJ_12482_249; **Location:** country: Malaysia; locality: Redang islands; **Identification:** identifiedBy: Loh KH and Du Jianguo

##### Notes

Harborne et al. 2000; s: *Pomacentrus gymmnorhynchus* Yusuf et al. 2001.

#### Pomacentrus lepidogenys

Fowler & Bean, 1928

E29B9232-2336-5CCB-912E-14AE969C20D1

##### Materials

- **Type status:** Other material. **Occurrence:** occurrenceID: BDJ_12482_250; **Location:** country: Malaysia; locality: Redang islands; **Identification:** identifiedBy: Loh KH and Du Jianguo

##### Notes

Harborne et al. 2000

#### Pomacentrus moluccensis

Bleeker, 1853

1B28B53C-67AA-52E4-B974-12E68D80441A

##### Materials

- **Type status:** Other material. **Occurrence:** occurrenceID: BDJ_12482_251; **Location:** country: Malaysia; locality: Redang islands; **Identification:** identifiedBy: Loh KH and Du Jianguo

##### Notes

Harborne et al. 2000; Yusuf et al. 2001; This study.

#### Pomacentrus philippinus

Evermann & Seale, 1907

ED6CC5DA-B8E9-535F-9BAD-87FFDABBA7C7

##### Materials

- **Type status:** Other material. **Occurrence:** occurrenceID: BDJ_12482_252; **Location:** country: Malaysia; locality: Redang islands; **Identification:** identifiedBy: Loh KH and Du Jianguo

##### Notes

Harborne et al. 2000; Yusuf et al. 2001.

#### Pomacentrus simsiang

Bleeker, 1856

ADC9DEA2-25AC-5E5A-B940-2510B231F8AA

##### Materials

- **Type status:** Other material. **Occurrence:** occurrenceID: BDJ_12482_253; **Location:** country: Malaysia; locality: Redang islands; **Identification:** identifiedBy: Loh KH and Du Jianguo

##### Notes

Newly recorded in Redang islands + This study.

#### Pomacentrus tripunctatus

Cuvier, 1830

6A56A554-12B7-5794-B63E-B15AE7AF44E1

##### Materials

- **Type status:** Other material. **Occurrence:** occurrenceID: BDJ_12482_254; **Location:** country: Malaysia; locality: Redang islands; **Identification:** identifiedBy: Loh KH and Du Jianguo

##### Notes

This study.

#### Stegestes lividus

(Forster, 1801)

38A643E4-EE21-51C6-BAD6-7477BFADA556

##### Materials

- **Type status:** Other material. **Occurrence:** occurrenceID: BDJ_12482_255; **Location:** country: Malaysia; locality: Redang islands; **Identification:** identifiedBy: Loh KH and Du Jianguo

##### Notes

s: *Stagestes lividusYusuf et al. 2001*.

#### Priacanthus blochii

Bleeker, 1853

ACE41B8D-8517-55BC-A829-476117DB6E33

##### Materials

- **Type status:** Other material. **Occurrence:** occurrenceID: BDJ_12482_256; **Location:** country: Malaysia; locality: Redang islands; **Identification:** identifiedBy: Loh KH and Du Jianguo

##### Notes

Harborne et al. 2000

#### Priacanthus hamrur

(Forsskål, 1775)

40D841FD-5405-5191-8569-08D6DF229141

##### Materials

- **Type status:** Other material. **Occurrence:** occurrenceID: BDJ_12482_257; **Location:** country: Malaysia; locality: Redang islands; **Identification:** identifiedBy: Loh KH and Du Jianguo

##### Notes

Harborne et al. 2000

#### Pseudochromis fuscus

Müller & Troschel, 1849

634AAEE7-41D3-5081-98E8-EC5654D99492

##### Materials

- **Type status:** Other material. **Occurrence:** occurrenceID: BDJ_12482_258; **Location:** country: Malaysia; locality: Redang islands; **Identification:** identifiedBy: Loh KH and Du Jianguo

##### Notes

s: *Pseudocromis fuscus* Yusuf et al. 2001.

#### Bolbometopon muricatum

(Valenciennes, 1840)

4EA63DED-9397-5F8B-9D1B-4FC72621CC73

##### Materials

- **Type status:** Other material. **Occurrence:** occurrenceID: BDJ_12482_259; **Location:** country: Malaysia; locality: Redang islands; **Identification:** identifiedBy: Loh KH and Du Jianguo

##### Ecological interactions

###### Conservation status

VU

##### Notes

Harborne et al. 2000; Yusuf et al. 2001; This study.

#### Chlorurus bleekeri

(de Beaufort, 1940)

9E7125CE-D281-52F1-888D-BE1D8553F11C

##### Materials

- **Type status:** Other material. **Occurrence:** occurrenceID: BDJ_12482_260; **Location:** country: Malaysia; locality: Redang islands; **Identification:** identifiedBy: Loh KH and Du Jianguo

##### Notes

s: *Scarus bleekeriYusuf et al. 2001*; This study.

#### Chlorurus capistratoides

(Bleeker, 1847)

0D08A982-D13E-5639-BBFB-AF8279CDE591

##### Materials

- **Type status:** Other material. **Occurrence:** occurrenceID: BDJ_12482_261; **Location:** country: Malaysia; locality: Redang islands; **Identification:** identifiedBy: Loh KH and Du Jianguo

##### Notes

Newly recorded in Redang islands + This study.

#### Chlorurus sordidus

(Forsskål, 1775)

A74C00C0-17EE-51DC-BC53-E98A363A3B78

##### Materials

- **Type status:** Other material. **Occurrence:** occurrenceID: BDJ_12482_262; **Location:** country: Malaysia; locality: Redang islands; **Identification:** identifiedBy: Loh KH and Du Jianguo

##### Notes

s: *Scarus sordidusYusuf et al. 2001*; This study.

#### Scarus caudofasciatus

(Günther, 1862)

DE0AAD35-871E-5B44-897A-37FE2F908561

##### Materials

- **Type status:** Other material. **Occurrence:** occurrenceID: BDJ_12482_263; **Location:** country: Malaysia; locality: Redang islands; **Identification:** identifiedBy: Loh KH and Du Jianguo

##### Notes

* Newly recorded in Malaysia + This study.

#### Scarus ghobban

Forsskål, 1775

7E6DF2D4-1867-51B5-9E07-716178D604CD

##### Materials

- **Type status:** Other material. **Occurrence:** occurrenceID: BDJ_12482_264; **Location:** country: Malaysia; locality: Redang islands; **Identification:** identifiedBy: Loh KH and Du Jianguo

##### Notes

Harborne et al. 2000; Yusuf et al. 2001; This study.

#### Scarus niger

Forsskål, 1776

041C7445-19A2-50C2-B8CF-3D46D8ADC7FA

##### Materials

- **Type status:** Other material. **Occurrence:** occurrenceID: BDJ_12482_265; **Location:** country: Malaysia; locality: Redang islands; **Identification:** identifiedBy: Loh KH and Du Jianguo

##### Notes

Harborne et al. 2000; Yusuf et al. 2001; This study.

#### Scarus prasiognathos

Valenciennes, 1840

42849EEC-60CC-5863-AAAC-27E18905B986

##### Materials

- **Type status:** Other material. **Occurrence:** occurrenceID: BDJ_12482_266; **Location:** country: Malaysia; locality: Redang islands; **Identification:** identifiedBy: Loh KH and Du Jianguo

##### Notes

s: *Scarus prasiognathus* Yusuf et al. 2001; This study.

#### Scarus psittacus

Forsskål, 1775

9CD86501-10D2-56A7-A7FF-7E0CDEF68C8E

##### Materials

- **Type status:** Other material. **Occurrence:** occurrenceID: BDJ_12482_267; **Location:** country: Malaysia; locality: Redang islands; **Identification:** identifiedBy: Loh KH and Du Jianguo

##### Notes

This study.

#### Scarus quoyi

Valenciennes, 1840

A432C35B-9F54-544D-BEF4-04D2AED3E915

##### Materials

- **Type status:** Other material. **Occurrence:** occurrenceID: BDJ_12482_268; **Location:** country: Malaysia; locality: Redang islands; **Identification:** identifiedBy: Loh KH and Du Jianguo

##### Notes

Yusuf et al. 2001; This study.

#### Scarus rivulatus

Valenciennes, 1840

E170A6CE-4BF1-5532-9B53-B31813091DCB

##### Materials

- **Type status:** Other material. **Occurrence:** occurrenceID: BDJ_12482_269; **Location:** country: Malaysia; locality: Redang islands; **Identification:** identifiedBy: Loh KH and Du Jianguo

##### Notes

Yusuf et al. 2001; This study.

#### Scarus rubroviolaceus

Bleeker, 1847

84D990EB-8A36-5503-AFF5-B48875EE4292

##### Materials

- **Type status:** Other material. **Occurrence:** occurrenceID: BDJ_12482_270; **Location:** country: Malaysia; locality: Redang islands; **Identification:** identifiedBy: Loh KH and Du Jianguo

##### Notes

Yusuf et al. 2001; This study.

#### Scarus schlegeli

(Bleeker, 1861)

D4E72501-A532-50EB-958A-936DCC24F57E

##### Materials

- **Type status:** Other material. **Occurrence:** occurrenceID: BDJ_12482_271; **Location:** country: Malaysia; locality: Redang islands; **Identification:** identifiedBy: Loh KH and Du Jianguo

##### Notes

Yusuf et al. 2001

#### Scarus tricolor

Bleeker, 1847

D5817498-F12F-5DAC-85A1-A9E9E33E7BAB

##### Materials

- **Type status:** Other material. **Occurrence:** occurrenceID: BDJ_12482_272; **Location:** country: Malaysia; locality: Redang islands; **Identification:** identifiedBy: Loh KH and Du Jianguo

##### Notes

Harborne et al. 2000

#### Scaridae sp.

29AB4688-9F0A-5E95-A889-CCC6865C54B0

##### Materials

- **Type status:** Other material. **Occurrence:** occurrenceID: BDJ_12482_273; **Location:** country: Malaysia; locality: Redang islands; **Identification:** identifiedBy: Harborne A, Fenner D, Barnes A, Beger M, Harding S, Roxburgh T

##### Notes

Harborne et al. 2000

#### Pterois russellii

Bennett, 1831

7C4635D6-A203-5A4D-A70B-B702FD2D4324

##### Materials

- **Type status:** Other material. **Occurrence:** occurrenceID: BDJ_12482_274; **Location:** country: Malaysia; locality: Redang islands; **Identification:** identifiedBy: Loh KH and Du Jianguo

##### Notes

This study.

#### Aethaloperca rogaa

(Forsskål, 1775)

E34535EF-8391-55AA-953F-876A5E2393C5

##### Materials

- **Type status:** Other material. **Occurrence:** occurrenceID: BDJ_12482_275; **Location:** country: Malaysia; locality: Redang islands; **Identification:** identifiedBy: Loh KH and Du Jianguo

##### Notes

Harborne et al. 2000

#### Cephalopholis boenack

(Bloch, 1790)

A2E8F641-A97E-5062-8816-5279955B80DF

##### Materials

- **Type status:** Other material. **Occurrence:** occurrenceID: BDJ_12482_276; **Location:** country: Malaysia; locality: Redang islands; **Identification:** identifiedBy: Loh KH and Du Jianguo

##### Notes

Harborne et al. 2000; Yusuf et al. 2001; This study.

#### Cephalopholis cyanostigma

(Valenciennes, 1828)

4F3CBE09-7A11-5711-9AF3-32455059F181

##### Materials

- **Type status:** Other material. **Occurrence:** occurrenceID: BDJ_12482_277; **Location:** country: Malaysia; locality: Redang islands; **Identification:** identifiedBy: Loh KH and Du Jianguo

##### Notes

Harborne et al. 2000; Yusuf et al. 2001; This study.

#### Cephalopholis formosa

(Shaw, 1812)

05DFD30B-5214-59ED-8667-8B1334AA6948

##### Materials

- **Type status:** Other material. **Occurrence:** occurrenceID: BDJ_12482_278; **Location:** country: Malaysia; locality: Redang islands; **Identification:** identifiedBy: Loh KH and Du Jianguo

##### Notes

Harborne et al. 2000; This study. s: *Cephalopholis formosus* Yusuf et al. 2001.

#### Cephalopholis microprion

(Bleeker, 1852)

17FFD960-E494-5176-A898-E96B560B3FDA

##### Materials

- **Type status:** Other material. **Occurrence:** occurrenceID: BDJ_12482_279; **Location:** country: Malaysia; locality: Redang islands; **Identification:** identifiedBy: Loh KH and Du Jianguo

##### Notes

Yusuf et al. 2001

#### Cephalopholis miniata

(Forsskål, 1775)

66C3EC7C-030F-5FC2-A80B-A4AEF8D6C072

##### Materials

- **Type status:** Other material. **Occurrence:** occurrenceID: BDJ_12482_280; **Location:** country: Malaysia; locality: Redang islands; **Identification:** identifiedBy: Loh KH and Du Jianguo

##### Notes

Harborne et al. 2000

#### Diploprion bifasciatum

Cuvier, 1828

460DEBE4-759E-5D8B-98DF-2CBA71D3B5A0

##### Materials

- **Type status:** Other material. **Occurrence:** occurrenceID: BDJ_12482_281; **Location:** country: Malaysia; locality: Redang islands; **Identification:** identifiedBy: Loh KH and Du Jianguo

##### Notes

Yusuf et al. 2001

#### Epinephelus fasciatus

(Forsskål, 1775)

2A0490FA-233A-5901-8768-4740E96491D4

##### Materials

- **Type status:** Other material. **Occurrence:** occurrenceID: BDJ_12482_282; **Location:** country: Malaysia; locality: Redang islands; **Identification:** identifiedBy: Loh KH and Du Jianguo

##### Notes

Harborne et al. 2000; This study. s: *Ephinephelus fasciatusYusuf et al. 2001*.

#### Epinephelus fuscoguttatus

(Forsskål, 1775)

A1489086-3D69-5A34-8938-87CE74087BFE

##### Materials

- **Type status:** Other material. **Occurrence:** occurrenceID: BDJ_12482_283; **Location:** country: Malaysia; locality: Redang islands; **Identification:** identifiedBy: Loh KH and Du Jianguo

##### Ecological interactions

###### Conservation status

NT

##### Notes

s: *Ephinephelus fuscoguttatus* Yusuf et al. 2001

#### Epinephelus ongus

(Bloch, 1790)

A95BBC05-D2A5-5BA1-AD10-99349241F51B

##### Materials

- **Type status:** Other material. **Occurrence:** occurrenceID: BDJ_12482_284; **Location:** country: Malaysia; locality: Redang islands; **Identification:** identifiedBy: Loh KH and Du Jianguo

##### Notes

s: *Ephinephelus ongus* Yusuf et al. 2001

#### Epinephelus polyphekadion

(Bleeker, 1849)

1FA319F3-5878-5570-BCA3-937D879D7E50

##### Materials

- **Type status:** Other material. **Occurrence:** occurrenceID: BDJ_12482_285; **Location:** country: Malaysia; locality: Redang islands; **Identification:** identifiedBy: Loh KH and Du Jianguo

##### Ecological interactions

###### Conservation status

NT

##### Notes

Harborne et al. 2000

#### Epinephelus quoyanus

(Valenciennes, 1830)

2D1B2346-43FD-5C7A-A40C-264430A1E3B0

##### Materials

- **Type status:** Other material. **Occurrence:** occurrenceID: BDJ_12482_286; **Location:** country: Malaysia; locality: Redang islands; **Identification:** identifiedBy: Loh KH and Du Jianguo

##### Notes

s: *Ephinephelus quoyanus* Yusuf et al. 2001.

#### Plectropomus areolatus

(Rüppell, 1830)

098255FA-04FB-52FA-9573-66DACEF3F7C5

##### Materials

- **Type status:** Other material. **Occurrence:** occurrenceID: BDJ_12482_287; **Location:** country: Malaysia; locality: Redang islands; **Identification:** identifiedBy: Loh KH and Du Jianguo

##### Ecological interactions

###### Conservation status

VU

##### Notes

s: *Plectropomus aerolatus* Harborne et al. 2000.

#### Plectropomus leopardus

(Lacepède, 1802)

0B9C3736-954B-515C-A3BF-1AA04CCB0214

##### Materials

- **Type status:** Other material. **Occurrence:** occurrenceID: BDJ_12482_288; **Location:** country: Malaysia; locality: Redang islands; **Identification:** identifiedBy: Loh KH and Du Jianguo

##### Ecological interactions

###### Conservation status

NT

##### Notes

Harborne et al. 2000; Yusuf et al. 2001; This study.

#### Plectropomus maculatus

(Bloch, 1790)

533B2F8F-4B46-5903-9DF5-1D6A2DEC4F5E

##### Materials

- **Type status:** Other material. **Occurrence:** occurrenceID: BDJ_12482_289; **Location:** country: Malaysia; locality: Redang islands; **Identification:** identifiedBy: Loh KH and Du Jianguo

##### Notes

Harborne et al. 2000; Yusuf et al. 2001.

#### Siganus argenteus

(Quoy & Gaimard, 1825)

9B9D2103-4B50-5412-BB71-81C20BE5DC3A

##### Materials

- **Type status:** Other material. **Occurrence:** occurrenceID: BDJ_12482_290; **Location:** country: Malaysia; locality: Redang islands; **Identification:** identifiedBy: Loh KH and Du Jianguo

##### Notes

Harborne et al. 2000

#### Siganus canaliculatus

(Park, 1797)

5EB16501-D5A8-53EE-BB29-2BC42CC17EFC

##### Materials

- **Type status:** Other material. **Occurrence:** occurrenceID: BDJ_12482_291; **Location:** country: Malaysia; locality: Redang islands; **Identification:** identifiedBy: Loh KH and Du Jianguo

##### Notes

Yusuf et al. 2001

#### Siganus corallinus

(Valenciennes, 1835)

2E02DD24-FA8B-5CBB-AB5A-C80932CEF0A7

##### Materials

- **Type status:** Other material. **Occurrence:** occurrenceID: BDJ_12482_292; **Location:** country: Malaysia; locality: Redang islands; **Identification:** identifiedBy: Loh KH and Du Jianguo

##### Notes

Harborne et al. 2000; Yusuf et al. 2001; This study.

#### Siganus guttatus

(Bloch, 1787)

F8FB66E0-AB52-568E-9C09-37ABA5748A1D

##### Materials

- **Type status:** Other material. **Occurrence:** occurrenceID: BDJ_12482_293; **Location:** country: Malaysia; locality: Redang islands; **Identification:** identifiedBy: Loh KH and Du Jianguo

##### Notes

Harborne et al. 2000; Yusuf et al. 2001; This study.

#### Siganus javus

(Linnaeus, 1766)

AE0FD580-0CF3-5BD7-9476-C7F27A1E3DC8

##### Materials

- **Type status:** Other material. **Occurrence:** occurrenceID: BDJ_12482_294; **Location:** country: Malaysia; locality: Redang islands; **Identification:** identifiedBy: Loh KH and Du Jianguo

##### Notes

This study.

#### Siganus puellus

(Schlegel, 1852)

2E6AC24A-4BF4-526D-A952-B833F1986DF0

##### Materials

- **Type status:** Other material. **Occurrence:** occurrenceID: BDJ_12482_295; **Location:** country: Malaysia; locality: Redang islands; **Identification:** identifiedBy: Loh KH and Du Jianguo

##### Notes

Harborne et al. 2000; Yusuf et al. 2001.

#### Siganus punctatus

(Schneider & Forster, 1801)

3B3277CA-8103-58C6-805B-B5BF9F8C1D34

##### Materials

- **Type status:** Other material. **Occurrence:** occurrenceID: BDJ_12482_296; **Location:** country: Malaysia; locality: Redang islands; **Identification:** identifiedBy: Loh KH and Du Jianguo

##### Notes

This study.

#### Siganus spinus

(Linnaeus, 1758)

CDE2CEB7-C726-5081-838A-B84B274B21AF

##### Materials

- **Type status:** Other material. **Occurrence:** occurrenceID: BDJ_12482_297; **Location:** country: Malaysia; locality: Redang islands; **Identification:** identifiedBy: Loh KH and Du Jianguo

##### Notes

Harborne et al. 2000

#### Siganus vermiculatus

(Valenciennes, 1835)

11ED2761-6AF6-590A-A02D-9F2820D481F5

##### Materials

- **Type status:** Other material. **Occurrence:** occurrenceID: BDJ_12482_298; **Location:** country: Malaysia; locality: Redang islands; **Identification:** identifiedBy: Loh KH and Du Jianguo

##### Notes

Harborne et al. 2000; Yusuf et al. 2001.

#### Siganus virgatus

(Valenciennes, 1835)

8CA16CA9-B3E3-5DB1-B381-96B919897187

##### Materials

- **Type status:** Other material. **Occurrence:** occurrenceID: BDJ_12482_299; **Location:** country: Malaysia; locality: Redang islands; **Identification:** identifiedBy: Loh KH and Du Jianguo

##### Notes

Harborne et al. 2000; Yusuf et al. 2001; This study.

#### Siganus vulpinus

(Schlegel & Müller, 1845)

1C56A3A0-746C-57C4-9E50-17AA1AD73277

##### Materials

- **Type status:** Other material. **Occurrence:** occurrenceID: BDJ_12482_300; **Location:** country: Malaysia; locality: Redang islands; **Identification:** identifiedBy: Loh KH and Du Jianguo

##### Notes

Harborne et al. 2000; This study. s: *Siganus vulpinis* Yusuf et al. 2001.

#### Sphyraena barracuda

(Edwards, 1771)

AD8B6C7F-E291-5DAD-9B54-FB3382323771

##### Materials

- **Type status:** Other material. **Occurrence:** occurrenceID: BDJ_12482_301; **Location:** country: Malaysia; locality: Redang islands; **Identification:** identifiedBy: Loh KH and Du Jianguo

##### Notes

Yusuf et al. 2001; This study.

#### Sphyraena flavicauda

Rüppell, 1838

6FF50E5D-CC50-5013-969A-F071F2B20366

##### Materials

- **Type status:** Other material. **Occurrence:** occurrenceID: BDJ_12482_302; **Location:** country: Malaysia; locality: Redang islands; **Identification:** identifiedBy: Loh KH and Du Jianguo

##### Notes

Harborne et al. 2000; This study.

#### Sphyraena jello

Cuvier, 1829

6D6FAFD1-8F38-5AB3-A2A9-359221F6A51E

##### Materials

- **Type status:** Other material. **Occurrence:** occurrenceID: BDJ_12482_303; **Location:** country: Malaysia; locality: Redang islands; **Identification:** identifiedBy: Loh KH and Du Jianguo

##### Notes

Harborne et al. 2000

#### Sphyraena obtusata

Cuvier, 1829

FEAA3355-E340-5898-9058-E065483E8EB2

##### Materials

- **Type status:** Other material. **Occurrence:** occurrenceID: BDJ_12482_304; **Location:** country: Malaysia; locality: Redang islands; **Identification:** identifiedBy: Loh KH and Du Jianguo

##### Notes

Newly recorded in Redang islands + This study.

#### Sphyraena qenie

Klunzinger, 1870

FE0C778D-A972-5FCB-B391-FDB6E89CB288

##### Materials

- **Type status:** Other material. **Occurrence:** occurrenceID: BDJ_12482_305; **Location:** country: Malaysia; locality: Redang islands; **Identification:** identifiedBy: Loh KH and Du Jianguo

##### Notes

s: *Sphyraena quenie* Harborne et al. 2000

#### Inimicus didactylus

(Pallas, 1769)

B1FEE2D1-6FD3-5BF9-8048-507D78417238

##### Materials

- **Type status:** Other material. **Occurrence:** occurrenceID: BDJ_12482_306; **Location:** country: Malaysia; locality: Redang islands; **Identification:** identifiedBy: Loh KH and Du Jianguo

##### Notes

This study.

#### Synodus variegatus

(Lacepède, 1803)

2935C3EE-154C-5DF3-BA79-BBEC3D720B2B

##### Materials

- **Type status:** Other material. **Occurrence:** occurrenceID: BDJ_12482_307; **Location:** country: Malaysia; locality: Redang islands; **Identification:** identifiedBy: Loh KH and Du Jianguo

##### Notes

Yusuf et al. 2001; This study.

#### Synodus sp.

BD44C77B-2970-5CBA-83E7-CF307C8CF3D9

##### Materials

- **Type status:** Holotype. **Occurrence:** occurrenceID: BDJ_12482_308; **Location:** country: Malaysia; locality: Redang islands; **Identification:** identifiedBy: Harborne A, Fenner D, Barnes A, Beger M, Harding S, Roxburgh T

##### Notes

Harborne et al. 2000

#### Arothron mappa

(Lesson, 1831)

B6C8AEBB-4D2F-5C74-88E1-7961F7CB2683

##### Materials

- **Type status:** Other material. **Occurrence:** occurrenceID: BDJ_12482_309; **Location:** country: Malaysia; locality: Redang islands; **Identification:** identifiedBy: Loh KH and Du Jianguo

##### Notes

Newly recorded in Redang islands + This study.

#### Arothron nigropunctatus

(Bloch & Schneider, 1801)

1389BFC8-BAE5-5206-8E75-718E3615F892

##### Materials

- **Type status:** Other material. **Occurrence:** occurrenceID: BDJ_12482_310; **Location:** country: Malaysia; locality: Redang islands; **Identification:** identifiedBy: Loh KH and Du Jianguo

##### Notes

Harborne et al. 2000; Yusuf et al. 2001; This study.

#### Arothron stellatus

(Anonymous, 1798)

2B3B3D2F-CD49-581B-83C5-C14638EA632B

##### Materials

- **Type status:** Other material. **Occurrence:** occurrenceID: BDJ_12482_311; **Location:** country: Malaysia; locality: Redang islands; **Identification:** identifiedBy: Loh KH and Du Jianguo

##### Notes

Harborne et al. 2000; Yusuf et al. 2001; This study.

#### Terapon jarbua

(Forsskål, 1775)

C721F0F7-9503-5427-A17C-A221E20C7B8C

##### Materials

- **Type status:** Other material. **Occurrence:** occurrenceID: BDJ_12482_312; **Location:** country: Malaysia; locality: Redang islands; **Identification:** identifiedBy: Loh KH and Du Jianguo

##### Notes

Yusuf et al. 2001

#### Helcogramma striata

Hansen, 1986

EB4A80EF-E780-5B74-ADE4-8F23579E1AB0

##### Materials

- **Type status:** Other material. **Occurrence:** occurrenceID: BDJ_12482_313; **Location:** country: Malaysia; locality: Redang islands; **Identification:** identifiedBy: Loh KH and Du Jianguo

##### Notes

This study.

#### Zanclus cornutus

(Linnaeus, 1758)

B6F296C9-4D50-58CE-8907-FE92085468BB

##### Materials

- **Type status:** Other material. **Occurrence:** occurrenceID: BDJ_12482_314

##### Notes

Harborne et al. 2000; s: *Zinclus cornutus* Yusuf et al. 2001.

## Discussion

Similar to our results, the most diverse family recorded in previous studies was Pomacentridae with 35 species (Harborne et al. 2000) and 37 species (Yusuf et al. 2001), followed by Labridae with 31 species (Harborne et al. 2000) and 22 species (Yusuf et al. 2001). However, Acanthuridae was reported to have relatively low species richness with only four species recorded by Harborne et al. (2000) and two by Yusuf et al. (2001). Surgeonfish were not observed in the Redang Islands by Comley et al. (2004) nor in the present study, even though the area had high coral coverage.

The CFDI value for the Redang Islands was 132 and, from this value, based on the proposed formula by Allen (1988) and Allen and Werner (2002), we estimated that there were 427 coral reef fish species in the Redang Islands Marine Park. However, only 314 species were observed, which is low compared with the estimated number of species. More surveys on different sites and in different seasons should be conducted in future research. A similar number of Chaetodontidae species were observed at the Redang Islands Marine Park as at the Perhentian, Tinggi Islands Marine Park and other marine parks in Peninsular Malaysia. However, fewer were observed compared with other areas in the Coral Triangle area, for example, the Tunku Abdul Rahman Park, Sabah (Townsend 2015).

According to the IUCN Red List, eight of the species recorded are Near Threatened (*Carcharhinus melanopterus*, *Chaetodon trifascialis*, *Choerodon schoenleinii*, *Epinephelus fuscoguttatus*, *E. polyphekadion*, *Plectropomus leopardus*, *Taeniura lymma* and *Triaenodon obesus*), eleven are Vulnerable (*Bolbometopon muricatum*, *Chaetodon trifasciatus, Chlorurus sordidusDascyllus trimaculatus, Epinephelus fuscoguttatus, E. polyphekadion, Halichoeres marginatus, Heniochus acuminatus, Nebrius ferrugineus, Neopomacentrus cyanomos* and *Plectropomus areolatus*), and three are Endangered (*Amphiprion clarkia, Cheilinus undulatus*, and *Scarus ghobban*). These species require futher attention in terms of park management practices and conservation issues (e.g. habitat integrity, anthropogenic impact and possible poaching within the park area).

## Figures and Tables

**Figure 2a.**
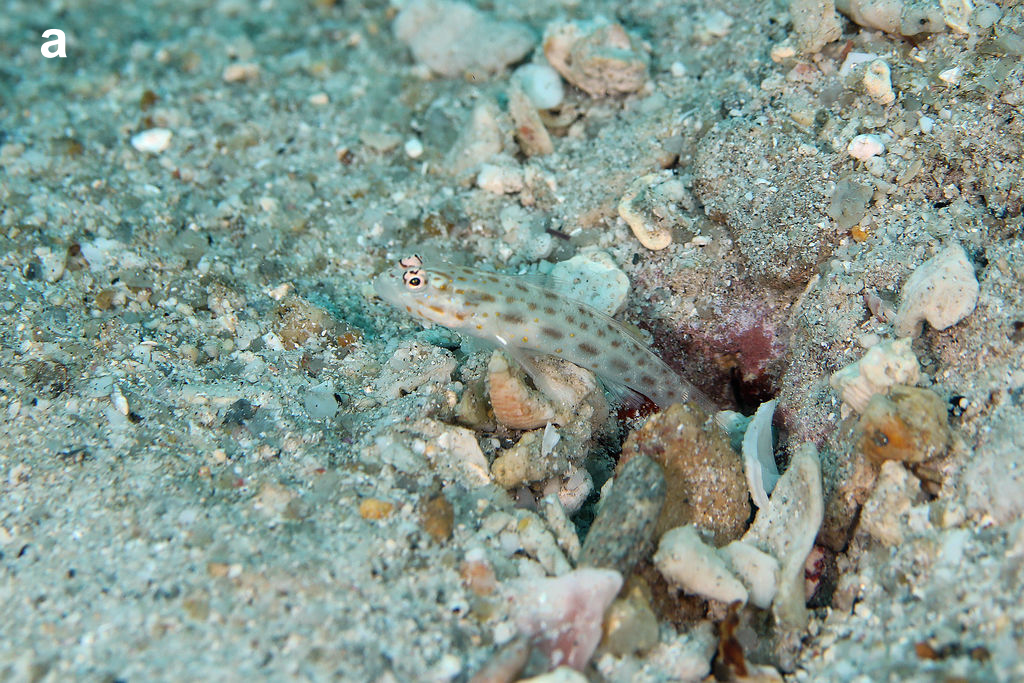
*Ctenogobiops mitodes*

**Figure 2b.**
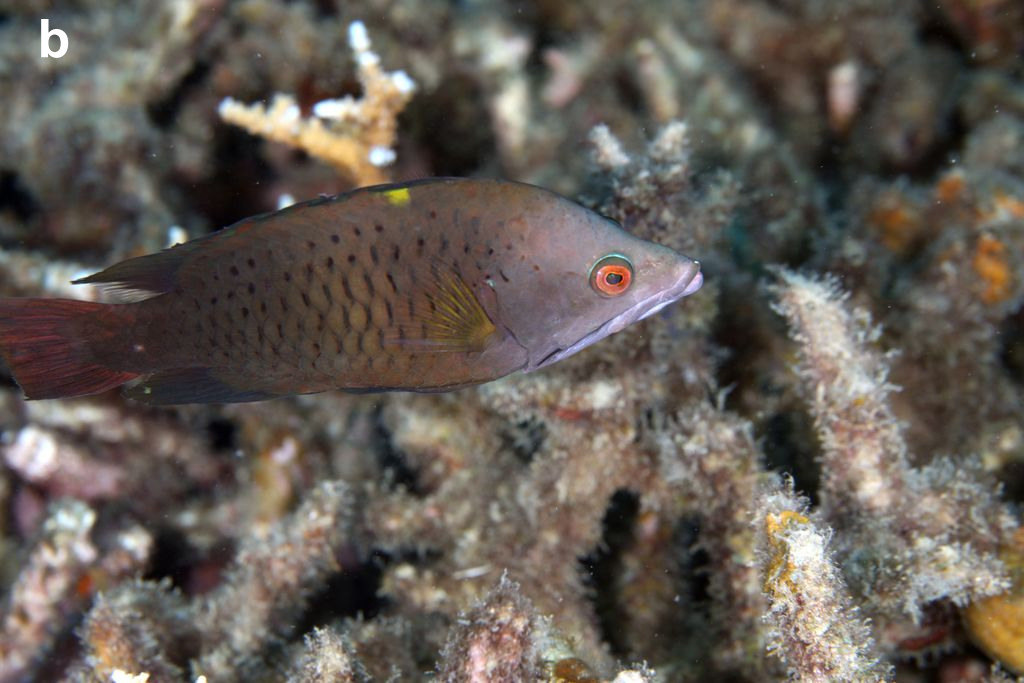
*Epibulus brevis* (male)

**Figure 2c.**
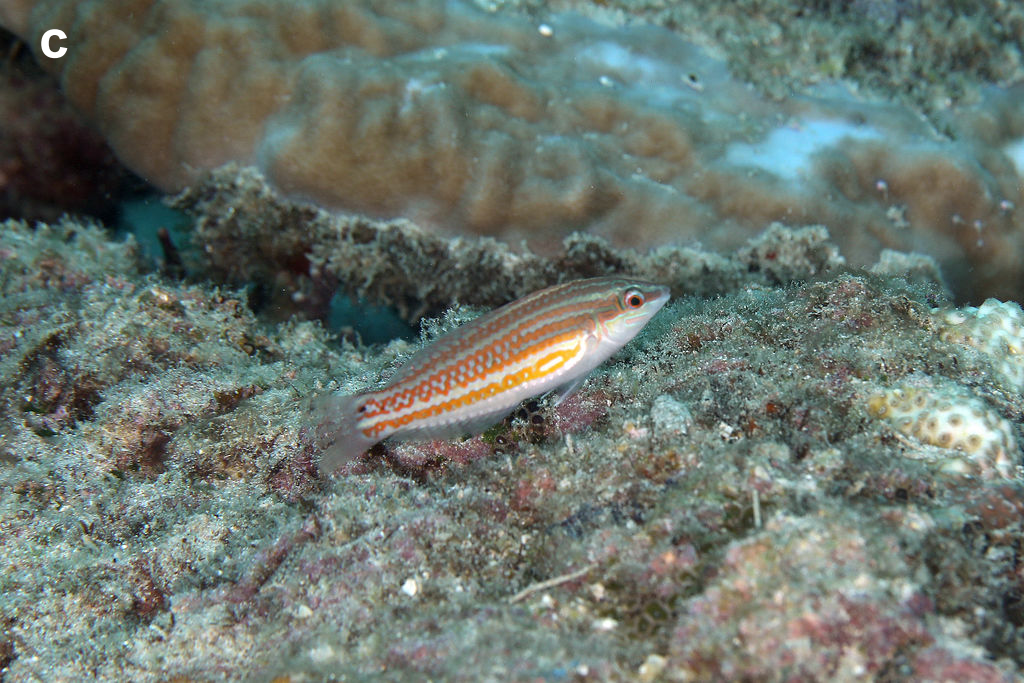
*Halichoeres erdmanni* (female)

**Figure 2d.**
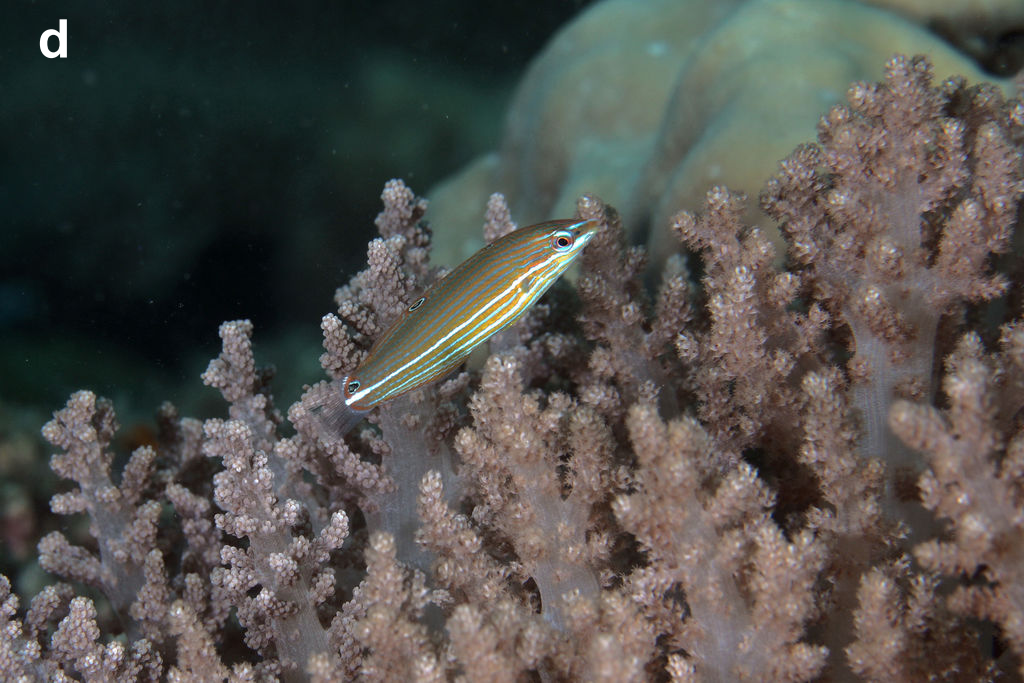
*Halichoeres richmondi*

**Figure 2e.**
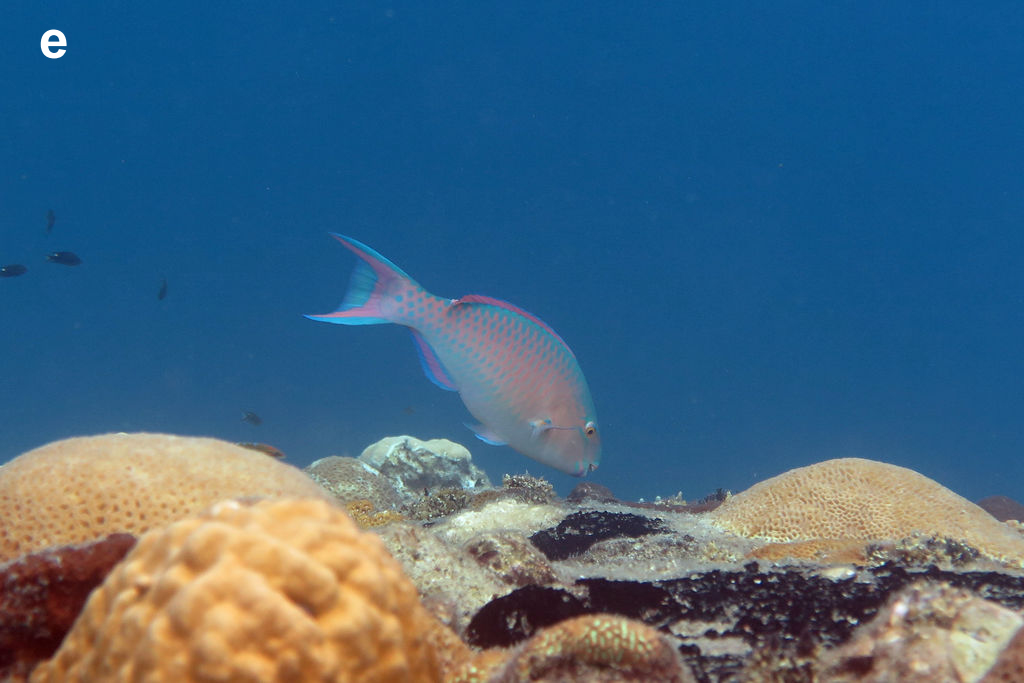
*Scarus caudofasciatus* (male)

**Table 1.** Checklist of marine fishes of Redang archipelago

Subclass, Order, Family, Genus and species	Habitat types	Threat to humans	IUCN status	Other remarks
Elasmobranchii				
Order Carcharhiniformes				
Family CARCHARHINIDAE (Requiem shark)				
*Carcharhinus melanopterus* (Quoy & Gaimard, 1824)	PEL, RFA	Traumatogenic	NT	[2000, 2001, 2019]
*Triaenodon obesus* (Rüppell, 1837)	PEL, RFA	Traumatogenic	NT	[2000]
Order Orectolobiformes				
Family GINGLYMOSTOMATIDAE (Nurse shark)				
*Nebrius ferrugineus* (Lesson, 1831)	CS, RFA	Traumatogenic	VU	[2000]
Order Myliobatiformes				
Family DASYATIDAE (Stingray)				
* Himantura * sp.				[2000]
*Taeniura lymma* (Forsskål, 1775)	RFA	Venomous	NT	[2000; 2001, 2011, 2019]
Actinopteri				
Order Acanthuriformes				
Family ACANTHURIDAE (Surgeonfish)				
*Acanthurus dussumieri* Valenciennes, 1835	SR, RFA		LC	[2000]
*Acanthurus lineatus* (Linnaeus, 1758)	SR, RFA	Venomous		[2000]
* Acanthurus * sp.			LC	[2001]
*Naso lituratus* (Forster, 1801)	SR, RFA	Venomous	LC	[2000, 2001]
*Naso unicornis* (Forsskål, 1775)	SR, RFA	Reports of ciguatera poisoning	LC	[2000]
Family POMACANTHIDAE (Angelfish)				
*Chaetodontoplus mesoleucus* (Bloch, 1787)	RFA		LC	[2000, 2019; 2001, s: *Chaetodontoplus mesoleucas*]
*Pomacanthus annularis* (Bloch, 1787)	SR, RFA		LC	[2000, 2001, 2011, 2019]
±*Pomacanthus imperator* (Bloch, 1787)	SR		LC	[2019]
*Pomacanthus sexstriatus* (Cuvier, 1831)	SR, RFA		LC	[2000, 2001, 2019]
*Pomacanthus xanthometopon* (Bleeker, 1853)	SR, RFA		LC	[2000]
Family SIGANIDAE (Rabbitfish)				
*Siganus argenteus* (Quoy & Gaimard, 1825)	SR, RFA	Venomous	LC	[2000]
*Siganus canaliculatus* (Park, 1797)	SR, RFA	Venomous	LC	[2001]
*Siganus corallinus* (Valenciennes, 1835)	SR, RFA	Venomous	LC	[2000, 2001, 2019]
*Siganus guttatus* (Bloch, 1787)	SR, RFA	Venomous	LC	[2000, 2001, 2019]
*Siganus javus* (Linnaeus, 1766)	SR, RFA	Venomous	DD	[2011, 2019]
*Siganus puellus* (Schlegel, 1852)	SR, RFA	Venomous	LC	[2000, 2001]
*Siganus punctatus* (Schneider & Forster, 1801)	SR, RFA	Venomous	LC	[2011, 2019]
*Siganus spinus* (Linnaeus, 1758)	SR, RFA	Venomous	LC	[2000]
*Siganus vermiculatus* (Valenciennes, 1835	SR, RFA	Venomous	LC	[2000, 2001]
*Siganus virgatus* (Valenciennes, 1835)	SR, RFA	Venomous	LC	[2000, 2001, 2019]
*Siganus vulpinus* (Schlegel & Müller, 1845)	SR, RFA	Venomous	LC	[2000, 2019; 2001, s: *Siganus vulpinis*]
Family ZANCLIDAE (Moorishn idol)				
*Zanclus cornutus* (Linnaeus, 1758)	SR, RFA		LC	[2000; 2001, s: *Zinclus cornutus*]
Order Acropomatiformes				
Family PEMPHERIDAE (Sweeper)				
*Pempheris oualensis* Cuvier, 1831	SR, RFA			[2000; 2019]
*Pempheris schwenkii* Cuvier, 1855	SR, RFA			[2000]
*Pempheris vanicolensis* Bleeker, 1877	SR, RFA			[2001]
Order Anguilliformes				
Family MURAENIDAE (Moray eel)				
±*Gymnothorax fimbriatus* (Bennett, 1832)	SR, RFA		LC	[2019]
*Gymnothorax javanicus* Bloch & Schneider, 1801	SR, RFA	Reports of ciguatera poisoning	LC	[2000, 2019]
Order Aulopiformes				
Family SYNODONTIDAE (Lizardfishes)				
*Synodus variegatus* (Lacepède, 1803)	CS, RFA		LC	[2001, 2019]
* Synodus * sp.				[2000]
Order Beloniformes				
Family HEMIRAMPHIDAE (Halfbeaks)				
* Hemiramphus * sp.				[2001]
*Hyporhamphus affinis* (Güther, 1866)	SR, PEL			[2001, s: *Hyporhampus affinis*]
Order Blenniiformes				
Family BLENNIIDAE (Blennies)				
±*Aspidontus taeniatus* Quoy & Gaimard, 1834	SR, RFA		LC	[2019]
*Atrosalarias fuscus* (Rüppell, 1838)	SR, RFA		LC	[2000]
*Ecsenius bicolor* (Day, 1888)	SR, RFA		LC	[2000, 2019]
*Ecsenius lineatus* Klausewitz, 1962	SR, RFA		LC	[2000]
*Ecsenius yaeyamaensis* (Aoyagi, 1954)	SR, RFA		LC	[2000]
*Meiacanthus grammistes* (Valenciennes, 1836)	SR, RFA	Venomous	LC	[2000]
*Plagoitremus rhinorhynchos* (Bleeker, 1852)	SR, RFA			[2001, s: *Plagoitremus rhinorhincus*]
* Salarias * sp.				[2001]
Family TRIPTERYGIIDAE (Threefin, triplefin blennies)				
*Helcogramma striata* Hansen, 1986	SR, RFA		LC	[2011, 2019]
Order Centrarchiformes				
Family KYPHOSIDAE (Rudderfish)				
*Kyphosus cinerascens* (Forsskål, 1775)	SR, RFA	Poisonous to eat	DD	[2001]
* Kyphosus * sp.				[2000]
Family TERAPONTIDAE (Grunts, tigerperches)				
*Terapon jarbua* (Forsskål, 1775)	CS, DEM		LC	[2001]
Order Gobiesociformes				
Family GOBIESOCIDAE (Clingfish)				
±*Diademichthys lineatus* (Sauvage, 1883)	SR, RFA		LC	[2019]
Order Gobiiformes				
Family GOBIIDAE (Gobies)				
±*Amblyeleotris rubrimarginata* Mohlmann & Randall, 2002	SR, RFA			[2019]
*Amblyeleotris steinitzi* (Klausewitz, 1974)	SR, RFA		LC	[2001]
*Amblygobius phalaena* (Valenciennes, 1837)	SR, RFA			[2001]
*Cryptocentrus cinctus* (Herre, 1936)	SR, RFA			[2000, 2001]
*Ctenogobiops pomastictus* Lubbock & Polunin, 1977	SR, RFA			[2001, s: *Ctenogobius pomastictus*]
*±*Ctenogobiops mitodes* Randall, Shao & Chen, 2007	SR, RFA			[2019]
±*Eviota sebreei* Jordan & Seale, 1906	SR, RFA		LC	[2019]
±*Exyrias bellissimus* (Smith, 1959)	SR, RFA		LC	[2019]
*Istigobius decoratus* (Herre, 1927)	SR, RFA		LC	[2001]
*Istigobius goldmanni* (Bleeker, 1852)	SR, RFA			[2011, 2019]
*Istigobius nigroocellatus* (Günther, 1873)	SR, RFA			[2011, 2019]
*Valenciennea longipinnis* (Lay & Bennett, 1839)	SR, RFA			[2001]
*Valenciennea muralis* (Valenciennes, 1837)	SR, RFA		LC	[2001]
*Valenciennea sexguttata* (Valenciennes, 1837)	SR, RFA		LC	[2001, 2019]
Family MICRODESMIDAE (Dartfish)				
*Gunnelichthys monostigma* Smith, 1958	SR, RFA			[2000]
Order Gonorynchiformes				
Family CHANIDAE (Milkfish)				
*Chanos chanos* (Forsskål, 1775)	CS, RFA			[2001]
Order Holocentriformes				
Family HOLOCENTRIDAE (Soldier, squirrelfish)				
±*Myripristis hexagona* (Lacepède, 1802)	SR, RFA		LC	[2019]
*Myripristis kuntee* Valenciennes, 1831.	SR, RFA		LC	[2001, s: *Myriptis kuntee*]
*Myripristis murdjan* (Forsskål, 1775)	SR, RFA	Reports of ciguatera poisoning	LC	[2000]
±*Sargocentron cornutum* (Bleeker, 1854)	SR, RFA	Venomous	LC	[2019]
*Sargocentron rubrum* (Forsskål, 1775)	SR, RFA	Venomous	LC	[2011, 2019]
*Sargocentron praslin* (Lacepède, 1802)	SR, RFA		LC	[2000]
* Sargocentron * sp.				[2001]
Order Mugiliformes				
Family MUGILIDAE (Mullet)				
*Crenemugil crenilabis* (Forsskål, 1775)	CS, RFA			[2001]
Order Perciformes *sedis mutablis*				
Family APOGONIDAE (Cardinalfishes)				
*Apogon aureus* (Lacepède, 1802)	SR, RFA			[2001]
*Cheilodipterus artus* Smith, 1961	SR, RFA			[2000]
*Cheilodipterus intermedius* Gon, 1993	SR, RFA			[2001]
*Cheilodipterus macrodon* (Lacepède, 1802)	SR, RFA		LC	[2000; 2001, m: *Cheilodipterus heptaxona*, s: *Cheilodipterus microdon*; 2019]
*Cheilodipterus quinquelineatus* Cuvier, 1828	SR, RFA			[2000, 2001, 2011, 2019]
*Ostorhinchus chrysopomus* (Bleeker 1854)	SR			[2001, s: *Apogon crysopomus*]
*Ostorhinchus compressus* (Smith & Radcliffe, 1911)	SR, RFA		LC	[2000, 2001, s: *Apogon compressus*; 2019]
*Ostorhinchus cookii* (Macleay, 1881)	SR, RFA		DD	[2000, s: *Ostorhinchus cookie*]
*Ostorhinchus cyanosoma* (Bleeker, 1853)	SR, RFA		LC	[2000, 2001, s: *Apogon cyanosoma*]
±*Ostorhinchus wassinki* (Bleeker, 1860)	SR, RFA			[2019]
*Pristicon rhodopterus* (Bleeker, 1852)	SR, RFA			[2001, s: *Apogon rhodopterus*]
*Taeniamia fucata* (Cantor, 1849)	SR, RFA		LC	[2000, 2001, s: *Archaima fucata*; 2019]
*Taeniamia macroptera* (Cuvier, 1828)	SR, RFA			[2001, s: *Archaima macroptera*; 2019]
*Taeniamia zosterophora* (Bleeker, 1856)	SR, RFA			[2000, s: *Archamia zoesterophora*; 2001, s: *Cheilodipterus zosterophora*; 2019]
Family CAESIONIDAE (Fusilier)				
*Caesio caerulaurea* Lacepède, 1801	SR, RFA		LC	[2000, 2001, 2019]
*Caesio cuning* (Bloch, 1791)	SR, RFA		LC	[2000]
*Caesio teres* Seale, 1906	SR, RFA		LC	[2000, 2019]
*Caesio xanthonota* Bleeker, 1853	SR, RFA		LC	[2001, s: *Caesio xanthonata*]
*Pterocaesio chrysozona* (Cuvier, 1830)	SR, RFA		LC	[2000]
*Pterocaesio marri* Schultz, 1953	SR, RFA		LC	[2000, 2019]
Family CARANGIDAE (Jack, pompanos)				
*Alepes melanoptera* (Swainson, 1839)	PEL		LC	[2011, 2019]
* Alepes * sp.				[2001]
*Carangoides bajad* (Forsskål, 1775)	SR, RFA		LC	[2000, 2019]
*Carangoides chrysophrys* (Cuvier, 1833)	SR, RFA			[2000, s: *Carangoides chrysophys*]
*Carangoides gymnostethus* (Cuvier, 1833)	SR, RFA		DD	[2000]
*Caranx melampygus* Cuvier, 1833	SR, RFA	Reports of ciguatera poisoning	LC	[2000]
*Caranx sexfasciatus* Quoy & Gaimard, 1825	SR, RFA			[2000]
*Caranx tille* Cuvier, 1833	SR, RFA			[2000]
*Elagatis bipinnulata* (Quoy & Gaimard, 1825)	SR, RFA	Reports of ciguatera poisoning	LC	[2000]
*Gnathanodon speciosus* (Forsskål, 1775)	SR, RFA		LC	[2000, 2001]
*Scomberoides lysan* (Forsskål, 1775)	SR, RFA		LC	[2001]
*Selaroides leptolepis* (Cuvier, 1833)	SR, RFA		LC	[2011, 2019]
*Trachinotus bailloni* (Lacepède, 1801)	SR, RFA		LC	[2000, 2001]
*Trachinotus blochii* (Lacepède, 1801)	SR, RFA	Reports of ciguatera poisoning	LC	[2000]
Family ECHENEIDAE (Remoras)				
*Echeneis naucrates* Linnaeus, 1758	RFA		DD	[2000, 2019]
Family EPHIPPIDAE (Spadefishes)				
*Platax teira* (Forsskål, 1775)	CS, RFA		LC	[2000, 2001, 2019]
* Platax * sp.				[2000]
Family GERREIDAE (Mojarrs)				
*Gerres oyena* (Forsskål, 1775)	CS, RFA		LC	[2001]
Family HAEMULIDAE (Sweetlips)				
*Plectorhinchus albovittatus* (Rüppell, 1838)	SR, RFA			[2000]
±*Pletorhinchus chaetodonoides* Lacepède, 1801	SR, RFA			[2019]
*Plectorhinchus chrysotaenia* (Bleeker, 1855)	SR, RFA			[2001, s: *Plectorhinchus celebicus*]
*Plectorhinchus gibbosus* (Lacepède, 1802)	SR, RFA	Reports of ciguatera poisoning	LC	[2000, 2001]
*Plectorhinchus lessonii* (Cuvier, 1830)	SR, RFA			[2000, s: *Plectorhinchus lessoni*]
*Plectorhinchus vittatus* (Linnaeus, 1758)	SR, RFA		LC	[2000]
Family LETHRINIDAE (Emperors)				
*Lethrinus erythropterus* Valenciennes, 1830	SR, RFA		LC	[2000, 2001, 2019]
*Lethrinus microdon* Valenciennes, 1830	SR, RFA	Reports of ciguatera poisoning	LC	[2000]
*Lethrinus olivaceus* Valenciennes, 1830	SR, RFA	Reports of ciguatera poisoning	LC	[2000, 2001]
*Lethrinus ornatus* Valenciennes, 1830	SR, RFA		LC	[2000]
* Lethrinus * sp.				[2001]
Family LUTJANIDAE (Snapper)				
*Lutjanus argentimaculatus* (Forsskål, 1775)	SR, RFA	Reports of ciguatera poisoning	LC	[2000, 2001]
*Lutjanus bohar* (Forsskål, 1775)	SR, RFA	Reports of ciguatera poisoning	LC	[2000]
*Lutjanus decussatus* (Cuvier, 1828)	SR, RFA		LC	[2001, 2019]
*Lutjanus fulviflamma* (Forsskål, 1775)	SR, RFA	Reports of ciguatera poisoning	LC	[2000]
*Lutjanus johnii* (Bloch, 1792)	SR, RFA		LC	[2000]
*Lutjanus kasmira* (Forsskål, 1775)	SR, RFA		LC	[2000]
*Lutjanus lutjanus* Bloch, 1790	SR, RFA		LC	[2000, 2001, 2019]
*Lutjanus quinquelineatus* (Bloch, 1790)	SR, RFA		LC	[2001, 2019]
*Lutjanus russellii* (Bleeker, 1849)	SR, RFA		LC	[2001, s: *Lutjanus russelli*; 2019]
*Lutjanus vitta* (Quoy & Gaimard, 1824)	SR, RFA		LC	[2000, 2001, 2019]
*Macolor niger* (Forsskål, 1775)	SR, RFA		LC	[2000, 2001]
Family MONODACTYLIDAE (Monos, Moonyfishes)				
*Monodactylus argenteus* (Linnaeus, 1758)	CS, PEL		DD	[2000, 2019; 2001, s: *Monodactylus argentius*]
Family MULLIDAE (Goatfish)				
*Mulloidichthys flavolineatus* (Lacepède, 1801)	SR, RFA		DD	[2000]
*Parupeneus ciliatus* (Lacepède, 1802)	SR, RFA		LC	[2000]
*Parupeneus indicus* (Shaw, 1803)	SR, RFA		LC	[2000]
*Upeneus tragula* Richardson, 1846	CS, RFA		LC	[2000, 2019]
Family NEMIPTERIDAE (Threadfin breams)				
*Pentapodus caninus* (Cuvier, 1830)	SR, RFA		LC	[2000, 2001]
*Pentapodus emeryii* (Richardson, 1843)	SR, RFA		LC	[2000]
*Scolopsis affinis* Peters, 1877	SR, RFA		LC	[2000, 2001, 2019].
*Scolopsis bilineata* (Bloch, 1793)	SR, RFA		LC	[2000, 2001, s: *Scolopsis bilineatus*; 2019]
*Scolopsis ciliata* (Lacepède, 1802)	SR, RFA		LC	[2000, 2001, s: *Scolopsis ciliates*; 2019]
*Scolopsis lineata* Quoy & Gaimard, 1824	SR, RFA		LC	[2000, 2001, s: *Scolopsis lineatus*; 2019]
*Scolopsis margaritifer* (Cuvier, 1830)	SR, RFA		LC	[2000, 2019; 2001, s: *Scolopsis margaritifera*]
*Scolopsis monogramma* (Cuvier, 1830)	SR, RFA		LC	[2000, 2001, 2011, 2019]
*Scolopsis trilineata* Kner, 1868	SR, RFA		LC	[2001, s: *Scolopsis trilineatus*]
*Scolopsis vosmeri* (Bloch, 1792)	SR, RFA		LC	[2000, 2001, 2019]
Family PRIACANTHIDAE (Bigeyes)				
*Priacanthus blochii* Bleeker, 1853	SR, RFA		LC	[2000]
*Priacanthus hamrur* (Forsskål, 1775)	SR, RFA		LC	[2000]
Family PSEUDOCHROMIDAE (Dottyback)				
*Pseudochromis fuscus* Müller & Troschel, 1849	SR, RFA		LC	[2001, s: *Pseudocromis fuscus*]
Family SERRANIDAE (Grouper)				
*Aethaloperca rogaa* (Forsskål, 1775).	SR, RFA		LC	[2000]
*Cephalopholis boenack* (Bloch, 1790)	SR, RFA			[2001, 2019]
*Cephalopholis cyanostigma* (Valenciennes, 1828)	SR, RFA		LC	[2001, 2019]
*Cephalopholis formosa* (Shaw, 1812)	SR, RFA		LC	[2001, 2019; 2001, s: *Cephalopholis formosus*]
*Cephalopholis microprion* (Bleeker, 1852)	SR, RFA		LC	[2001]
*Cephalopholis miniata* (Forsskål, 1775)	SR, RFA		LC	[2000]
*Diploprion bifasciatum* Cuvier, 1828	SR, RFA		LC	[2001]
*Epinephelus fasciatus* (Forsskål, 1775)	SR, RFA	Reports of ciguatera poisoning	LC	[2000, 2019; 2001, s: *Ephinephelus fasciatus*]
*Epinephelus fuscoguttatus* (Forsskål, 1775)	SR, RFA	Reports of ciguatera poisoning	VU	[2001, s: *Ephinephelus fuscoguttatus*]
*Epinephelus ongus* (Bloch, 1790)	SR, RFA		LC	[2001, s: *Ephinephelus ongus*]
*Epinephelus polyphekadion* (Bleeker, 1849)	SR, RFA	Reports of ciguatera poisoning	VU	[2000]
*Epinephelus quoyanus* (Valenciennes, 1830)	SR, RFA	Reports of ciguatera poisoning	LC	[2001, s: *Ephinephelus quoyanus*; 2019]
*Plectropomus areolatus* (Rüppell, 1830)	SR, RFA	Reports of ciguatera poisoning	VU	[2000, s: *Plectropomus aerolatus*]
*Plectropomus leopardus* (Lacepède, 1802)	SR, RFA	Reports of ciguatera poisoning	LC	[2000, 2001, 2019]
*Plectropomus maculatus* (Bloch, 1790)	SR, RFA	Reports of ciguatera poisoning	LC	[2000, 2001]
Order Perciformes				
Family CHAETODONTIDAE (Butterflyfish)				
*Chaetodon adiergastos* Seale, 1910	SR, RFA		LC	[2000]
*Chaetodon auriga* Forsskål, 1775	SR, RFA		LC	[2000]
*Chaetodon baronessa* Cuvier, 1829	SR, RFA		LC	[2000, 2001, 2019]
*Chaetodon lineolatus* Cuvier, 1831	SR, RFA		LC	[2000]
*Chaetodon lunulatus* Quoy & Gaimard, 1825	SR, RFA		LC	[2001]
*Chaetodon octofasciatus* Bloch, 1787	SR, RFA		LC	[2000, 2001, 2019]
*Chaetodon trifascialis* Quoy & Gaimard, 1825	SR, RFA		NT	[2000]
*Chaetodon trifasciatus* Park, 1797	SR, RFA		VU	[2000]
*Chaetodon wiebeli* Kaup, 1863	SR, RFA		LC	[2000, 2001]
*Chelmon rostratus* (Linnaeus, 1758)	SR, RFA		LC	[2000, 2019; 2001, s: *Chelmon rostratum*]
*Coradion chrysozonus* (Cuvier, 1831)	SR, RFA		LC	[2000, 2001, 2019]
*Heniochus acuminatus* (Linnaeus, 1758)	SR, RFA		VU	[2000, s: *Heniochus accuminatu*; 2001, s: *Heniochus acuminiatus*]
*Heniochus varius* (Cuvier, 1829)	SR, RFA		LC	[2000]
Family LABRIDAE (Wrasse)				
*Bodianus diana* (Lacepède, 1801)	SR, RFA		LC	[2000]
*Bodianus mesothorax* (Bloch & Schneider, 1801)	SR, RFA		LC	[2000, 2001]
*Cheilinus chlorourus* (Bloch, 1791)	SR, RFA		LC	[2000, 2019; 2001, s: *Cheilinus clorourus*]
*Cheilinus fasciatus* (Bloch, 1791)	SR, RFA		LC	[2000, 2001, 2019]
*Cheilinus trilobatus* Rüppell, 1835	SR, RFA		LC	[2000, 2001, 2019]
*Cheilinus undulatus* Rüppell, 1835	SR, RFA	Reports of ciguatera poisoning	EN	[2001]
*Choerodon schoenleinii* (Valenciennes, 1839)	SR, RFA		NT	[2000]
*Diproctacanthus xanthurus* (Bleeker, 1856)	SR, RFA		LC	[2000, 2001, 2019]
*±*Epibulus brevis* Carlson, Randall & Dawson, 2008	SR, RFA		LC	[2019]
*Epibulus insidiator* (Pallas, 1770)	SR, RFA	Reports of ciguatera poisoning	LC	[2000, 2001]
*Gomphosus varius* Lacepède, 1801	SR, RFA		LC	[2000, 2001, 2019]
±*Halichoeres bicolor* (Bloch & Schneider, 1801)	SR, RFA		LC	[2019]
*Halichoeres biocellatus* Schultz, 1960	SR, RFA		LC	[2000]
*Halichoeres chloropterus* (Bloch, 1791)	SR, RFA		LC	[2000, 2001]
*Halichoeres dussumieri* (Bloch & Schneider, 1801)	SR, RFA		LC	[2001]
*±*Halichoeres erdmanni* Randall & Allen, 2010	SR, RFA			[2019]
*Halichoeres hortulanus* (Lacepède, 1801)	SR, RFA		LC	[2001, 2019]
*Halichoeres leucurus* (Walbaum, 1792)	SR, RFA		LC	[2000, s: *Halichoeres purpurascens*; 2019]
*Halichoeres marginatus* Rüppell, 1835]	SR, RFA		VU	[2000, 2001]
*Halichoeres melanochir* Fowler & Bean, 1928	SR, RFA		LC	[2000]
*Halichoeres melanurus* (Bleeker, 1851)	SR, RFA		LC	[2001]
±*Halichoeres nebulosus* (Valenciennes, 1839)	SR, RFA		LC	[2019]
*Halichoeres prosopeion* (Bleeker, 1853)	SR, RFA		LC	[2000]
*±*Halichoeres richmondi* Fowler & Bean, 1928	SR, RFA		LC	[2019]
*Halichoeres scapularis* (Bennett, 1832)	SR, RFA		LC	[2000, 2001].
*Halichoeres vrolikii* (Bleeker, 1855)	SR, RFA		LC	[2000]
*Hemigymnus melapterus* (Bloch, 1791)	SR, RFA		LC	[2000, 2001, 2019]
*Labrichthys unilineatus* (Guichenot, 1847)	SR, RFA		LC	[2000, 2001]
*Labroides dimidiatus* (Valenciennes, 1839)	SR, RFA		LC	[2000, 2001, 2019]
*Leptojulis cyanopleura* (Bleeker, 1853)	SR, RFA		LC	[2000]
*Macropharyngodon meleagris* (Valenciennes, 1839)	SR, RFA		LC	[2000]
*Oxycheilinus celebicus* (Bleeker, 1853)	SR, RFA		LC	[2000]
*Oxycheilinus digramma* (Lacepède, 1801)	SR, RFA		LC	[2000, 2001, s: *Oxychelinius diagrammus*; 2019]
*Oxycheilinus mentalis* (Rüppell, 1828)	SR, RFA		LC	[2001, s: *Oxychelinius mentalis*]
*Oxycheilinus orientalis* (Günther, 1862)	SR, RFA		LC	[2000]
* Oxycheilinus * sp.	SR, RFA			[2001, s: *Oxychelinius* sp]
*Paracheilinus filamentosus* Allen, 1974	SR, RFA		LC	[2000, s: *Paracheilinus filamentous*]
*Pseudocheilinus evanidus J*ordan & Evermann, 1903	SR, RFA		LC	[2000, s: *Pseudocheilinus evanide*]
*Pteragogus cryptus* Randall, 1981	SR, RFA		LC	[2000, 2001]
*Stethojulis bandanensis* (Bleeker, 1851)	SR, RFA		LC	[2000]
±*Stethojulis interrupta* (Bleeker, 1851)	SR, RFA		LC	[2019]
*Stethojulis trilineata* (Bloch & Schneider, 1801)	SR, RFA		LC	[2000, 2001, 2019]
*Thalassoma hardwicke* (Bennett, 1830)	SR, RFA		LC	[2000]
*Thalassoma lunare* (Linnaeus, 1758)	SR, RFA		LC	[2001, 2019]
Family PINGUIPEDIDAE (Sandperch)				
*Parapercis snyderi* Jordan & Starks, 1905	SR, RFA			[2000, 2019]
*Parapercis xanthozona* (Bleeker, 1849)	SR, RFA			[2000, 2019]
Family POMACENTRIDAE (Damselfish)				
*Abudefduf bengalensis* (Bloch, 1787)	SR, RFA		LC	[2001, 2019]
*Abudefduf notatus* (Day, 1870)	SR, RFA		LC	[2000, 2001]
*Abudefduf septemfasciatus* (Cuvier, 1830)	SR, RFA			[2000]
*Abudefduf sexfasciatus* (Lacepède, 1801)	SR, RFA			[2001, 2019]
*Abudefduf sordidus* (Forsskål, 1775)	SR, RFA		LC	[2001, 2019]
*Abudefduf vaigiensis* (Quoy & Gaimard, 1825)	SR, RFA	Reports of ciguatera poisoning	LC	[2000, 2001, 2019]
*Amblyglyphidodon aureus* (Cuvier, 1830)	SR, RFA		LC	[2000, 2019]
*Amblyglyphidodon curacao* (Bloch, 1787)	SR, RFA		LC	[2000, 2001, 2019]
*Amblyglyphidodon leucogaster* (Bleeker, 1847)	SR, RFA		LC	[2000, 2001, 2019]
* Amblyglyphidodon * sp.				[2001]
*Amphiprion clarkii* (Bennett, 1830)	SR, RFA		EN	[2000, 2001, 2019]
*Amphiprion frenatus* Brevoort, 1856	SR, RFA		LC	[2000, 2001, 2019]
*Amphiprion melanopus* Bleeker, 1852	SR, RFA		LC	[2001]
*Amphiprion ocellaris* Cuvier, 1830	SR, RFA			[2000, 2001, 2019]
*Amphiprion perideraion* Bleeker, 1855	SR, RFA			[2000, 2001, s: *Amphiprion periderion*; 2019]
*Cheiloprion labiatus* (Day, 1870)	SR, RFA			[2000, 2001]
*Chromis atripectoralis* Welander & Schultz, 1951	SR, RFA			[2000, 2019; 2001, s: *Cromis atripectoralis*]
*Chromis lepidolepis* Bleeker, 1877 .	SR, RFA			[2000, s: *Chromis lepidolepsis*]
*Chromis ternatensis* (Bleeker, 1856)	SR, RFA			[2000]
±*Chromis viridis* (Cuvier, 1830)	SR, RFA			[2019]
*Chromis weberi* Fowler & Bean, 1928	SR, RFA			[2000, s: *Chromis weberii*]
*Chrysiptera leucopoma* (Cuvier, 1830)	SR, RFA			[2001]
*Dascyllus reticulatus* (Richardson, 1846)	SR, RFA			[2000, 2019; 2001, s: *Dascyllus reticulates*]
*Dascyllus trimaculatus* (Rüppell, 1829)	SR, RFA		VU	[2000, 2001, 2019]
*Dischistodus chrysopoecilus* (Bleeker, 1858)	SR, RFA			[2001, s: *Dischistodus chrysopaecilus*]
*Dischistodus melanotus* (Bleeker, 1858)	SR, RFA			[2000, 2001, 2019]
*Dischistodus perspicillatus* (Cuvier, 1830)	SR, RFA			[2000, 2019; 2001, s: *Dischistodus perspiciliatus*]
*Hemiglyphidodon plagiometopon* (Bleeker, 1852)	SR, RFA			[2000, s: *Hemiglyphidodon plagiometapon*; 2001, s: *Hemiglyphidodon plagiometopodon*]
*Neoglyphidodon melas* (Valenciennes, 1830)	SR, RFA			[2000, 2001, 2019]
*Neoglyphidodon nigroris* (Cuvier, 1830)	SR, RFA			[2001]
*Neoglyphidodon oxyodon* (Bleeker, 1858)	SR, RFA			[2000]
*Neoglyphidodon thoracotaeniatus* (Fowler & Bean, 1928)	SR, RFA			[2000]
*Neopomacentrus anabatoides* (Bleeker, 1847)	SR, RFA			[2000, 2001]
*Neopomacentrus azysron* (Bleeker, 1877)	SR, RFA			[2000, s: *Neopomacentrus azyros*]
*Neopomacentrus cyanomos* (Bleeker, 1856)	SR, RFA		VU	[2000, 2019; 2001, s: *Neopomacentrus cyanomus*]
*Neopomacentrus violascens* (Bleeker, 1848)	SR, RFA			[2001]
*Plectroglyphidodon lacrymatus* (Quoy & Gaimard 1825)	SR, RFA			[2000, 2001]
*Pomacentrus alexanderae* Evermann & Seale, 1907	SR, RFA			[2000, 2019; 2001, s: *Pomacentrus alexandrae*]
±*Pomacentrus armillatus* Allen, 1993	SR, RFA			[2019]
*Pomacentrus bankanensis* Bleeker, 1854	SR, RFA			[2001]
*Pomacentrus bintanensis* Allen, 1999	SR, RFA			[2001]
*Pomacentrus chrysurus* Cuvier, 1830	SR, RFA			[2000, 2001]
*Pomacentrus coelestis* Jordan & Starks, 1901	SR, RFA			[2000, s: *Pomacentrus coelestris*; 2001, 2019]
*Pomacentrus grammorhynchus* Fowler, 1918	SR, RFA			[2000; 2001, s: *Pomacentrus gymmnorhynchus*]
*Pomacentrus lepidogenys* Fowler & Bean, 1928 .	SR, RFA			[2000]
*Pomacentrus moluccensis* Bleeker, 1853	SR, RFA			[2000, 2001, 2019]
*Pomacentrus philippinus* Evermann & Seale, 1907	SR, RFA			[2000, 2001]
±*Pomacentrus simsiang* Bleeker, 1856	SR, RFA			[2019]
*Pomacentrus tripunctatus* Cuvier, 1830	SR, RFA			[2011, 2019]
*Stegestes lividus* (Forster, 1801)	SR, RFA			[2001, s: *Stagestes lividus*]
Family SCARIDAE (Parrotfish)				
*Bolbometopon muricatum* (Valenciennes, 1840)	SR, RFA		VU	[2000, 2001, 2019]
*Chlorurus bleekeri* (de Beaufort, 1940)	SR, RFA		LC	[2001, s: *Scarus bleekeri*; 2019]
±*Chlorurus capistratoides* (Bleeker, 1847)	SR, RFA		LC	[2019]
*Chlorurus sordidus* (Forsskål, 1775)	SR, RFA	Reports of ciguatera poisoning	VU	[2001, s: *Scarus sordidus*; 2019]
*±*Scarus caudofasciatus* (Günther, 1862)	SR, RFA		LC	[2019]
*Scarus ghobban* Forsskål, 1775	SR, RFA		EN	[2000, 2001, 2019]
*Scarus niger* Forsskål, 1775	SR, RFA		LC	[2000, 2001, 2019]
*Scarus prasiognathos* Valenciennes, 1840	SR, RFA		LC	[2001, s: *Scarus prasiognathus*; 2019]
*Scarus psittacus* Forsskål, 1775	SR, RFA		DD	[2011, 2019]
*Scarus quoyi* Valenciennes, 1840	SR, RFA		LC	[2001, 2019]
*Scarus rivulatus* Valenciennes, 1840	SR, RFA		LC	[2001, 2019]
*Scarus rubroviolaceus* Bleeker, 1847	SR, RFA		LC	[2001, 2019]
*Scarus schlegeli* (Bleeker, 1861)	SR, RFA		LC	[2001]
*Scarus tricolor* Bleeker, 1847	SR, RFA		LC	[2000]
* Scaridae * sp.				[2000]
Family SCORPAENIDAE (Scoprionfish)				
*Pterois russellii* Bennett, 1831	SR, RFA	Venomous		[2011, 2019]
Family SPHYRAENIDAE (Barracuda)				
*Sphyraena barracuda* (Edwards, 1771)	SR, RFA	Traumatogenic	LC	[2000, 2019]
*Sphyraena flavicauda* Rüppell, 1838	SR, RFA		LC	[2000, 2019]
*Sphyraena jello* Cuvier, 1829	SR, RFA	Reports of ciguatera poisoning	LC	[2000]
±*Sphyraena obtusata* Cuvier, 1829	SR, RFA		LC	[2019]
*Sphyraena qenie* Klunzinger, 1870	SR, RFA		LC	[2000, s: *Sphyraena quenie*]
Family SYNANCEIIDAE (Stonefish)				
*Inimicus didactylus* (Pallas, 1769).	CS, RFA	Venomous		[2011, 2019]
Order Syngnathiformes				
Family CENTRISCIDAE (Shrimpfish, Razorfish)				
*Aeoliscus strigatus* (Günther, 1861)	SR, RFA		DD	[2001]
Order Tetraodontiformes				
Family BALISTIDAE (Triggerfish)				
*Balistapus undulatus* (Park, 1797)	SR, RFA	Traumatogenic.		[2000]
*Balistoides viridescens* (Bloch & Schneider, 1801)	SR, RFA	Reports of ciguatera poisoning		[2000; 2001, s: *Balistiodes viridescens*]
*Melichthys vidua* (Richardson, 1845)	SR, RFA			[2001]
*Pseudobalistes flavimarginatus* (Rüppell, 1829)	SR, RFA	Reports of ciguatera poisoning		[2000; 2001]
*Sufflamen bursa* (Bloch & Schneider, 1801)	SR, RFA			[2000]
Family BELONIDAE (Needlefish)				
*Strongylura incisa* (Valenciennes, 1846)	SR, RFA			[2000]
*Tylosurus crocodilus* (Péron & Lesueur, 1821)	SR, RFA	Traumatogenic	LC	[2001]
Family DIODONTIDAE (Porcupinefish)				
*Diodon hystrix* Linnaeus, 1758	RFA	Poisonous to eat	LC	[2000, 2019]
*Diodon liturosus* Shaw, 1804	RFA	Reports of ciguatera poisoning		[2000, 2019]
Family MONACANTHIDAE (Filefish, Leatherjacket)				
*Aluterus monoceros* (Linnaeus, 1758)	SR, RFA	Reports of ciguatera poisoning	LC	[2000, s: *Aluterus monoceres*; 2019]
*Aluterus scriptus* (Osbeck, 1765)	SR, RFA	Reports of ciguatera poisoning	LC	[2000; 2001, s: *Aluteres scriptus*]
*Cantherhines dumerilii* (Hollard, 1854)	SR, RFA		LC	[2000]
Family OSTRACIIDAE (Squared)				
*Ostracion cubicus* Linnaeus, 1758	SR, RFA.		LC	[2000, 2001, 2019]
Family TETRAODONTIDAE (Pufferfish)				
±*Arothron mappa* (Lesson, 1831)	SR, RFA	Poisonous to eat	LC	[2019]
*Arothron nigropunctatus* (Bloch & Schneider, 1801)	SR, RFA	Poisonous to eat	LC	[2000, 2001, 2019]
*Arothron stellatus* (Anonymous, 1798)	SR, RFA	Poisonous to eat	LC	[2000, 2001, 2019]
